# GlnA3*_Mt_* is able to glutamylate spermine but it is not essential for the detoxification of spermine in *Mycobacterium tuberculosis*

**DOI:** 10.1128/jb.00439-24

**Published:** 2025-01-30

**Authors:** Sergii Krysenko, Carine Sao Emani, Moritz Bäuerle, Maria Oswald, Andreas Kulik, Christian Meyners, Doris Hillemann, Matthias Merker, Gareth Prosser, Inken Wohlers, Felix Hausch, Heike Brötz-Oesterhelt, Agnieszka Mitulski, Norbert Reiling, Wolfgang Wohlleben

**Affiliations:** 1 Interfaculty Institute of Microbiology and Infection Medicine Tübingen (IMIT), Department of Microbiology and Biotechnology, University of Tübingen 9188 , Tübingen, Baden-Württemberg, Germany; 2 Cluster of Excellence 'Controlling Microbes to Fight Infections', University of Tübingen 9188 , Tübingen, Baden-Württemberg, Germany; 3 Microbial Interface Biology, Research Center Borstel, Leibniz Lung Center 28413 , Borstel, Schleswig-Holstein, Germany; 4 Institute of Organic Chemistry and Biochemistry, Technical University Darmstadt, Darmstadt, Hessen, Germany; 5 National Reference Center for Mycobacteria, Research Center Borstel, Leibniz Lung Center 28413 , Borstel, Schleswig-Holstein, Germany; 6 Evolution of the Resistome, Research Center Borstel, Leibniz Lung Center 28413 , Borstel, Schleswig-Holstein, Germany; 7 Data Science, Research Center Borstel, Leibniz Lung Center 28413 , Borstel, Schleswig-Holstein, Germany; 8 Centre for Synthetic Biology, Technical University of Darmstadt 26536 , Darmstadt, Hessen, Germany; 9 Interfaculty Institute of Microbiology and Infection Medicine Tübingen (IMIT), Department of Microbial Bioactive Compounds, University of Tübingen 9188 , Tübingen, Baden-Württemberg, Germany; 10 German Center for Infection Research (DZIF), Partner Site Tübingen, Tübingen, Germany; 11 German Center for Infection Research (DZIF), Partner Site Hamburg-Lübeck-Borstel-Riems, Borstel, Germany; University of Illinois ChicagoPharmaceutical Sciences, Center for Biomolecular Sciences, Chicago, Illinois, USA

**Keywords:** GS-like enzyme, GlnA3, glutamylation, polyamine metabolism, tuberculosis, infection, Rv3065

## Abstract

*Mycobacterium tuberculosis* is well adapted to survive and persist in the infected host, escaping the host’s immune response. Since polyamines such as spermine, which are synthesized by infected macrophages, are able to inhibit the growth of *M. tuberculosis*, the pathogen needs strategies to cope with these toxic metabolites. The actinomycete *Streptomyces coelicolor*, a close relative of *M. tuberculosis,* makes use of a gamma-glutamylation pathway to functionally neutralize spermine. We therefore considered whether a similar pathway would be functional in *M. tuberculosis*. In the current study, we demonstrated that *M. tuberculosis* growth was inhibited by the polyamine spermine. Using *in vitro* enzymatic assays we determined that GlnA3*_Mt_* (Rv1878) possesses genuine gamma-glutamylspermine synthetase catalytic activity. We further showed that purified His-Strep-GlnA3*_Mt_,* as well as native GlnA3*_Mt_,* prefer spermine as a substrate over putrescine, cadaverine, spermidine, or other monoamines and amino acids, suggesting that GlnA3*_Mt_* may play a specific role in the detoxification of the polyamine spermine. However, the deletion of the *glnA3* gene in *M. tuberculosis* did not result in growth inhibition or enhanced sensitivity of *M. tuberculosis* in the presence of high spermine concentrations. Gene expression analysis of spermine-treated *M. tuberculosis* revealed no difference in the level of *glnA3_Mt_* expression relative to untreated cells, whereas a gene encoding a previously characterized efflux pump (Mmr; *rv3065*) was significantly upregulated. This suggests that bacterial survival under elevated spermine concentrations can not only be achieved by detoxification of spermine itself but also by mechanisms resulting in decreased spermine levels in the bacteria.

**IMPORTANCE:**

Upon *Mycobacterium tuberculosis* infection macrophages synthesize the polyamine spermine, which at elevated concentrations is toxic for *M. tuberculosis*. Based on our investigations of spermine resistance in the closely related actinomycete *Streptomyces coelicolor*, we hypothesized that the glutamylspermine synthetase GlnA3 may be responsible for the resistance of *M. tuberculosis* against toxic spermine. Here we show that GlnA3_*Mt*_ can indeed covalently modify spermine via glutamylation. However, GlnA3_*Mt*_ is probably not the only resistance mechanism since a *glnA3* null mutant of *M. tuberculosis* can survive under spermine stress. Gene expression studies suggest that an efflux pump may participate in resistance. Thus a combination of GlnA3_*Mt*_ and specific efflux pumps acting as putative spermine transporters may constitute an active spermine-detoxification system in *M. tuberculosis*.

## INTRODUCTION

Worldwide, tuberculosis (TB) remains the most prevalent, persisting, and difficult-to-treat infectious disease and is associated with high mortality. In 2022, an estimated 10.6 million people developed TB disease, with an estimated 1.3 million deaths (1). Tuberculosis is caused by pathogenic mycobacteria of the *Mycobacterium tuberculosis* complex, which are well adapted to survive and persist within infected patients (2). An overall increase in the global burden of multidrug-resistant (MDR) and rifampicin-resistant (RR) TB severely jeopardizes control of the TB epidemic as envisaged by the WHO End TB strategy. MDR/RR-TB has been identified on all continents with approximately 410,000 cases reported in 2022 (1). Treatment of MDR*-*TB infections is of particular difficulty due to the extended duration, poor safety, and high costs involved. Several antibiotics are effective in treating MDR-TB infections; however, these drugs often show limited efficacy, and their use is coupled with diverse and dose-limiting side effects. Thus, the identification of pathways that are essential for mycobacterial growth *in vivo* would provide new targets for the rational design of more effective anti-TB agents that could be active against MDR-TB.

Polyamines are small aliphatic polyvalent cations, predominantly derived from amino acids such as ornithine, arginine, and lysine (3). They are widely distributed in nature and are present in all organisms, with the most common cellular polyamines being putrescine, cadaverine, spermidine, and spermine. Polyamines have been implicated in a wide range of biological processes and are synthesized in a linear pathway from putrescine via spermidine to spermine from the amino acids methionine, ornithine, lysine, and arginine (4). *De novo* biosynthesis of spermine has been reported in *Streptomyces* (5) and *Mycobacterium* (6, 7). Intracellular polyamines are synthesized in a very low concentration and are needed predominantly to stabilize the DNA and RNA in cells (7). In particular, *M. tuberculosis* strains express the spermidine synthase encoding gene *speE* (*Rv2601*).

Intracellular polyamine levels are elevated predominantly during exposure to various stress conditions (3, 8). Thus, intracellular polyamine concentrations are tightly regulated by cellular metabolic pathways (8), as polyamine excess has been proven toxic for prokaryotic and eukaryotic organisms and can lead to cell death (9–12). Polyamines are able to interact with negatively charged molecules like RNA, DNA, proteins, polyphosphate, and phospholipids (13). Consequently, an imbalance in polyamine metabolism can significantly affect cellular homeostasis. An excess of polyamines can be detoxified by modifications such as glutamylation. Detoxification is also the first step in subsequent polyamine assimilation as carbon and nitrogen sources under nutrient-limiting conditions. Polyamine catabolism has been investigated in several bacterial species revealing that the polyamine utilization pathway is not universal for all bacteria. This process has been studied extensively in Gram-negative bacteria such as *Escherichia coli* and *Pseudomonas aeruginosa* POA1. Polyamine utilization was reported to occur via the aminotransferase pathway (14), the γ-glutamylation pathway (14–16), the direct oxidation pathway for putrescine, and the spermine/spermidine dehydrogenase pathway. *E. coli* and *Bacillus subtilis* were shown to acetylate spermidine (16–19), whereas in the actinobacterial model organism *Streptomyces coelicolor*, the γ-glutamylation pathway was demonstrated (20–22).

*S. coelicolor* was shown to synthesize two functional, glutamine synthetase-like (GS-like) enzymes for polyamine glutamylation: the gamma-glutamylpolyamine synthetases GlnA2*_Sc_* and GlnA3*_Sc_*. GlnA3*_Sc_* was reported to be the central enzyme for the γ-glutamylation pathway in *S. coelicolor* and is highly specific for spermine and spermidine (20, 22). In *S. coelicolor*, GlnA3*_Sc_* permits detoxification and subsequent utilization of polyamines as a nitrogen source and is indispensable for bacterial survival under high polyamine concentrations (20). Recent *in silico* analysis of *glnA*-like genes across the actinobacterial phylum revealed numerous orthologues (*glnA2*, *glnA3,* and *glnA4*) that code for proteins potentially involved in the colonization, persistence, and survival of bacteria across diverse habitats (21). We have recently developed a series of inhibitors, specifically acting on GlnA4 (23) indicating that this group of enzymes can be selectively targeted.

Little is known about polyamine utilization and its regulation in *M. tuberculosis*. In *M. tuberculosis*, GlnA3*_Mt_* has been annotated and classified as “GS-like” (24, 25). While the function, regulation, and involvement of the glutamine synthetase (GS*_Mt_*) (GlnA1, Rv2220) in mycobacterial pathogenesis have been studied previously (26), the function of GS-like enzymes, such as GlnA2*_Mt_* (Rv2222c), GlnA3*_Mt_* (Rv1878), and GlnA4*_Mt_* (Rv2860c) so far remains unclear. Because many steps of nitrogen metabolism in *S. coelicolor* are nearly identical to those of *M. tuberculosis* and the function of the GS-like enzymes has been characterized (4, 27, 28) we hypothesized that *M. tuberculosis* might also exert a similar capacity for polyamine detoxification. In *S. coelicolor*, GlnA3*_Mt_* has been demonstrated to be the key enzyme for polyamine detoxification. The current study is the first attempt to investigate polyamine utilization in *M. tuberculosis* and the role of GlnA3*_Mt_* in this process.

## RESULTS

### GlnA3*_Mt_* from *M. tuberculosis* restores the growth of an *S. coelicolor glnA3* mutant in a polyamine-containing medium

As reported previously, GlnA3*_Sc_* confers the ability of *S. coelicolor* M145 to utilize polyamines as a sole nitrogen source (20). In order to test for a similar predicted function of the GlnA3*_Mt_* enzyme, the *glnA3_Mt_* gene was used for heterologous complementation of the *S. coelicolor* M145 Δ*glnA3_Sc_* mutant. The integrative vector pRM4 carrying a single copy of the *glnA3_Mt_* gene was integrated into the genome of the *S. coelicolor* M145 Δ*glnA3_Sc_* mutant. Successful integration of the construct in the *S. coelicolor* M145 genome was confirmed by PCR and sequencing. The phenotype of the complemented Δ*glnA3_Sc_* mutant was analyzed on complex, nitrogen-replete media (LB-agar, R5-agar), as well as on defined Evans-agar supplemented with single nitrogen sources (ammonium, L-glutamine, or the polyamines putrescine, spermidine, spermine). Growth and morphology of the parental *S. coelicolor* M145 strain, the Δ*glnA3_Sc_* mutant, and the Δ*glnA3_Sc_* complemented with *glnA3_Sc_* or *glnA3_Mt_* were monitored on solid media in a time range between 3 and 12 days of incubation at 30°C. The strains grew well on complex media (LB and R5) and the defined Evans medium supplemented with ammonium and glutamine (Table 1). The Δ*glnA3_Sc_* mutant was not able to grow on the defined Evans medium with polyamines as the only nitrogen source as previously reported (20). Heterologous complementation of the Δ*glnA3_Sc_* mutant with the *glnA3_Mt_* gene partially restored its growth on the defined Evans medium supplemented with either putrescine, spermidine, or spermine as the only nitrogen source (Table 1), thus demonstrating the functional equivalence of GlnA3*_Sc_* and GlnA3*_Mt_*.

**TABLE 1:** Physiological role of the *glnA3_Mt_* gene product in the *S. coelicolor glnA3* mutant grown in the presence of polyamines and other nitrogen sources^*a*^

Medium	M145 (WT)	*∆glnA3*_*Sc*_	*∆glnA3*_*Sc*_ *+glnA3*_*Mt*_	*∆glnA3*_*Sc*_ *+glnA3*_*Sc*_
LB-Agar	+++	+++	+++	+++
R5-Agar	+++	+++	+++	+++
Evans-Agar + NH_4_Cl (50 mM)	+++	+++	+++	+++
Evans-Medium + Glutamine (50 mM)	+++	+++	+++	+++
Evans-Medium + Putrescine (200 mM)	+++	−	+	++
Evans-Medium + Spermidine (25 mM)	+++	−	+	++
Evans-Medium + Spermine (25 mM)	+++	−	+	++

^*a*^ +: Poor growth; ++: moderate growth; +++: strong growth; −: no growth.

### GlnA3*_Mt_* catalyzes gamma-glutamylation of spermine and spermidine *in vitro*

**Fig 1:**
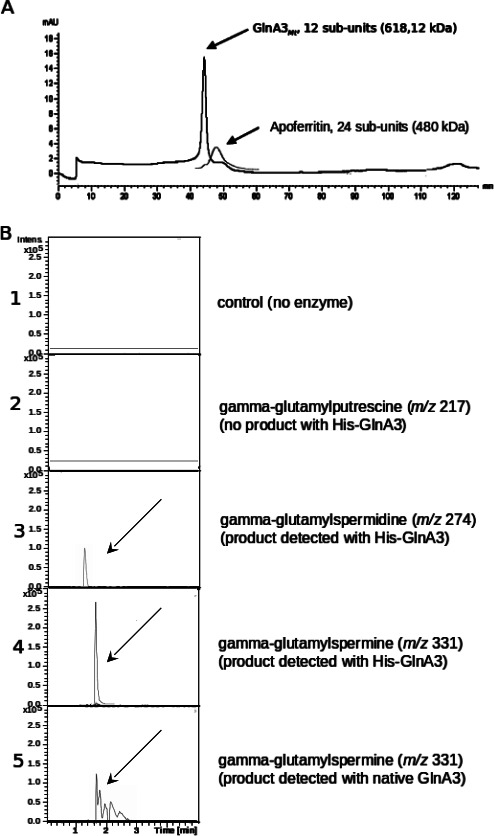
Purification of GlnA3*_Mt_* and generation of glutamylated spermine and spermidine by GlnA3*_Mt_* in an *in vitro* assay. (**A**) purification of GlnA3*_Mt_* by size-exclusion chromatography (SEC). SEC supported the putative dodecameric quaternary structure of the enzyme. A commercial apoferritin standard was used as a control for molecular mass estimation (elution profile shown in part). (**B**) HPLC/ESI-MS chromatograms demonstrating the presence or absence of glutamylated putrescine, spermidine, and spermine in a sample without (1) and with His-Strep-tagged GlnA3*_Mt_* enzyme (2–4). Gamma-glutamylputrescine (*m/z* 217) (2), gamma-glutamylspermidine (*m/z* 274) (3), gamma-glutamylspermine (*m/z* 331) (4). Reaction containing recombinant GlnA3*_Mt_* following removal of the Strep-His affinity tag, gamma-glutamylspermine (*m/z* 331) (5).

To elucidate the biochemical function of GlnA3*_Mt_* and to confirm that GlnA3*_Mt_* is able to catalyze the predicted glutamylation reaction of organic polyamines, we applied a previously validated high-performance liquid chromatography/mass spectrometry (HPLC/MS)-based assay (22). For this purpose, recombinant *glnA3_Mt_* was purified from *Escherichia coli* BL21 as a His- and Strep-tag fusion protein, with subsequent removal of the affinity tags. Analysis of the final product by size-exclusion chromatography supported the predicted dodecameric quaternary structure of GlnA3*_Mt_* (Fig. 1). The catalytic activity of purified GlnA3*_Mt_* was then tested by incubation with various polyamines in relevant reaction mixtures and the products analyzed using HPLC/MS analysis in negative MS mode (Fig. 1, reaction educts and products and their respective masses are depicted in Fig. S1). Our data showed that GlnA3*_Mt_* accepted glutamate and polyamines as substrates in an ATP-dependent manner. A reaction product with the expected *m/z* of 331 for glutamylspermine was detected, supporting the hypothesis that GlnA3*_Mt_* functions as a gamma-glutamylspermine synthetase (Fig. 1). Interestingly, no glutamylated putrescine or cadaverine reaction products and only a minor peak for glutamylated spermidine were detected, suggesting that GlnA3*_Mt_* possesses a high substrate affinity for spermine.

### Spermine is a preferred substrate for His-Strep-GlnA3*_Mt_*

**Fig 2:**
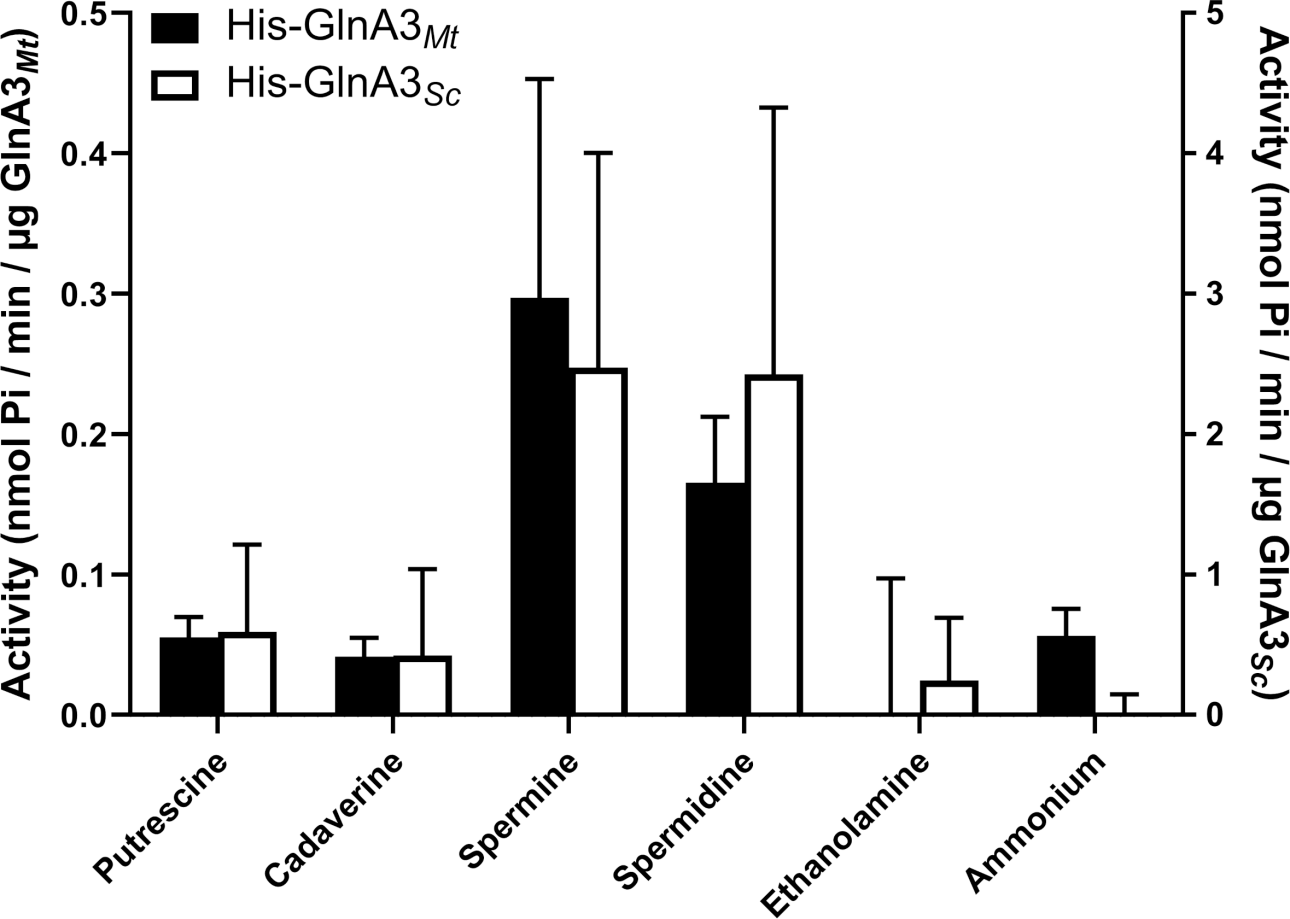
Specific activity of His-GlnA3*_Mt_* and His-GlnA3*_Sc_* with different nitrogen-containing substrates. All substrates were used at 50 mM. The mean value of *n* = 3 biological replicates with *n* = 3 technical replicates each with standard error is shown.

To study the substrate specificity of GlnA3*_Mt_* and its kinetic parameters, an adapted GS activity assay (29) based on the detection of inorganic phosphate (Pi) (released from ATP hydrolysis) was employed. Using this assay, both GlnA3*_Mt_* and its homolog GlnA3*_Sc_* were assessed for their ability to accept various amines (polyamines, monoamines, ammonium, and amino acids) as substrates (Fig. 2), as well as determining their relative catalytic rate with each. The results revealed that while GlnA3*_Mt_* can accept a variety of polyamines as substrates, the highest activity was observed with spermine and spermidine.

These findings were similar for the homologous protein His-GlnA3*_Sc_* from *S. coelicolor*, which also accepts various polyamines (Fig. 2) and possesses the highest turnover rate when using spermine or spermidine as substrates. Interestingly, the observed catalytic rates for GlnA3*_Sc_* were ~10-fold higher than those for GlnA3*_Mt_* with all substrates.

These results demonstrated a spermine-specific gamma-glutamylation activity of His-Strep-GlnA3*_Mt_* from *M. tuberculosis*, which was not observed for its homolog His-GlnA3*_Sc_* from *S. coelicolor*. In order to exclude the putative effects of the tags on the obtained results, we independently assessed the activity of cleaved GlnA3*_Mt_* with regard to the usage of putrescine, cadaverine, spermidine, and spermine as substrates. The enzyme was purified as a fusion protein with the SUMO-Tag, which was subsequently removed by cleavage. The enzymatic activity of the cleaved GlnA3*_Mt_* version was tested and compared with the His-Strep-GlnA3*_Mt_*. Although the cleaved GlnA3*_Mt_* revealed overall higher enzymatic activity than the tagged version in *in vitro* assays, the cleaved GlnA3*_Mt_* variant showed significantly higher activity with spermine compared to other polyamine substrates (Fig. S2). The effect remained the same after 1 h of additional incubation.

### GlnA3*_Mt_*, a predicted gamma-glutamylpolyamine synthetase structurally resembles GlnA3*_Sc_*

GlnA3*_Mt_* shares 52% amino acid similarity across the full-length sequence to GlnA3*_Sc_* from *S. coelicolor* M145 suggesting that the two orthologues may also share a similar tertiary structure (20). In order to study the structural properties of GlnA3*_Mt_* in the absence of experimental data, a homology modeling approach was used. The cloud-based software SWISS-MODEL (30) was used to generate an *in silico* 3D model of the protein, based on the available amino acid sequence and a template model; in this case, the previously solved structure of GlnA1*_Mt_* was used (31–34).

The GlnA3*_Mt_* model structure of *M. tuberculosis* comprises 12 identical sub-units (Fig. S3A) organized in two rings, with six sub-units in each (Fig. S3B) and one active site per sub-unit. Similar to both the solved GlnA*_Mt_* structure (32) and the GlnA3*_Sc_* model (20), a tunnel-like cavity is observed in each active site of GlnA3*_Mt_* (Fig. S3B and C) comprising individual substrate binding sites for ATP, glutamate, and ammonium. To improve confidence in the *in silico* data, we generated further models of GlnA3*_Mt_* using 20 different templates originating from various phyla of actinobacteria, proteobacteria, apicomplexa, and cyanobacteria. Model quality was then assessed based on GMQE, QMEAN, and MolProbity scores (35), and Ramachandran analysis (Fig. S3D) (36, 37). Structure prediction was afterward verified based on Alphafold predictions. The Alphafold prediction of the monomers matched the homology model monomer generated by the Chimera software.

**Fig 3:**
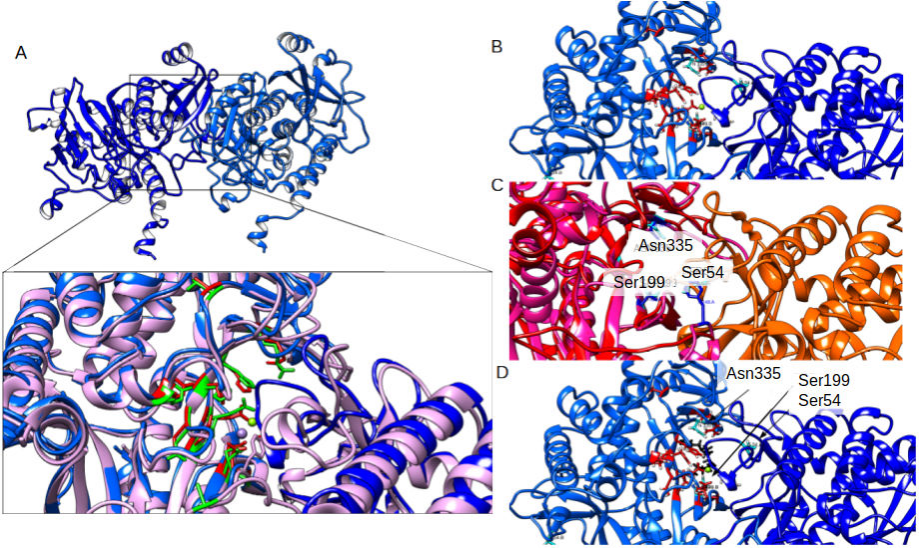
(**A**) Structural alignment of the 3D model structure of the GlnA3*_Mt_* enzyme (sub-units [**A] and [B**], the active site in-between). Based on and superposed with the GlnA*_Mt_* template (PDB code 1BVC) (27). Superposition of the GlnA3*_Sc_* (violet) and GlnA3*_Mt_* (blue) models with depicted key amino acids identified in GlnA3*_Sc_* (green, from reference [16]) and GlnA3*_Mt_* (red) (see Table 2). (**B**) Constellation of amino acid residues required for polyamine substrate binding in GlnA3*_Mt_* (red). (**C**) The residues selected for site-directed mutagenesis, i.e., Ser54, Ser199, and Asn335 (cyan) are depicted in the active site of GlnA3*_Mt_* among key residues in the binding pocket. (**D**) structural alignment of structure models of two sub-units of GlnA3*_Mt_* (red and orange) with the docked molecule of spermine (black) in the best-scored dock position overlaid with the crystal structure of PauA7*_Pa_* (violet).

The high diversity of templates used allowed the identification of conserved areas between the GlnA3*_Mt_* structure and other bacterial homologs. Across all analyzed proteins it was observed that most of the conserved amino acid residues were located in the active site of each monomer, namely in the glutamate, metal ions, and ATP binding sites. The active site of GlnA3*_Mt_* contains 9 out of 12 conserved amino acid residues at the ammonium binding site compared to the described gamma-glutamylpolyamine synthetase GlnA3*_Sc_*, although possessing more space on the loop for polyamine binding (no A169 residue in GlnA3*_Mt_*) (Fig. 3; Table 2).

**TABLE 2:** Superposition of residues in GlnA3*_Sc_* and GlnA3*_Mt_*

GlnA3*_Sc_*	E151	E153	E207	E214	H263	N260	G261	R316	H265	R339	W327	A169
GlnA3*_Mt_*	E137	E139	E190	E197	H246	S243	G244	R299	H248	R322	A310	None

### Site-directed mutagenesis of GlnA3*_Mt_* defines structural features important for spermine binding

In order to study the role of distinct residues of GlnA3*_Mt_* in catalytic activity and substrate recognition, a site-directed mutagenesis approach was undertaken. This approach relied on molecular docking with spermine and structural/sequence alignments with the available crystal structure of the gamma-glutamyl(mono-)polyamine synthetase from *P. aeruginosa* PauA7*_Pa_* (38), which is a functional homolog of GlnA3*_Mt_*. The comparison enabled the identification of potential key amino acids in the active site of GlnA3*_Mt_*. For example, Ser54 is part of the loop closing the active site, which is essential for substrate stabilization (38). In addition, Ser199 is a part of the so-called Tyr loop which partly forms the tunnel-like structure of the active site. Ser199 may also be part of the catalytic triad in GlnA3*_Mt_,* however, this is yet to be defined. Finally, Asn335 forms part of a second loop structure that closes the active site (Fig. 3A and C). The molecular modeling revealed that spermine may form interactions with those identified amino acid residues (Fig. 3B).

**Fig 4:**
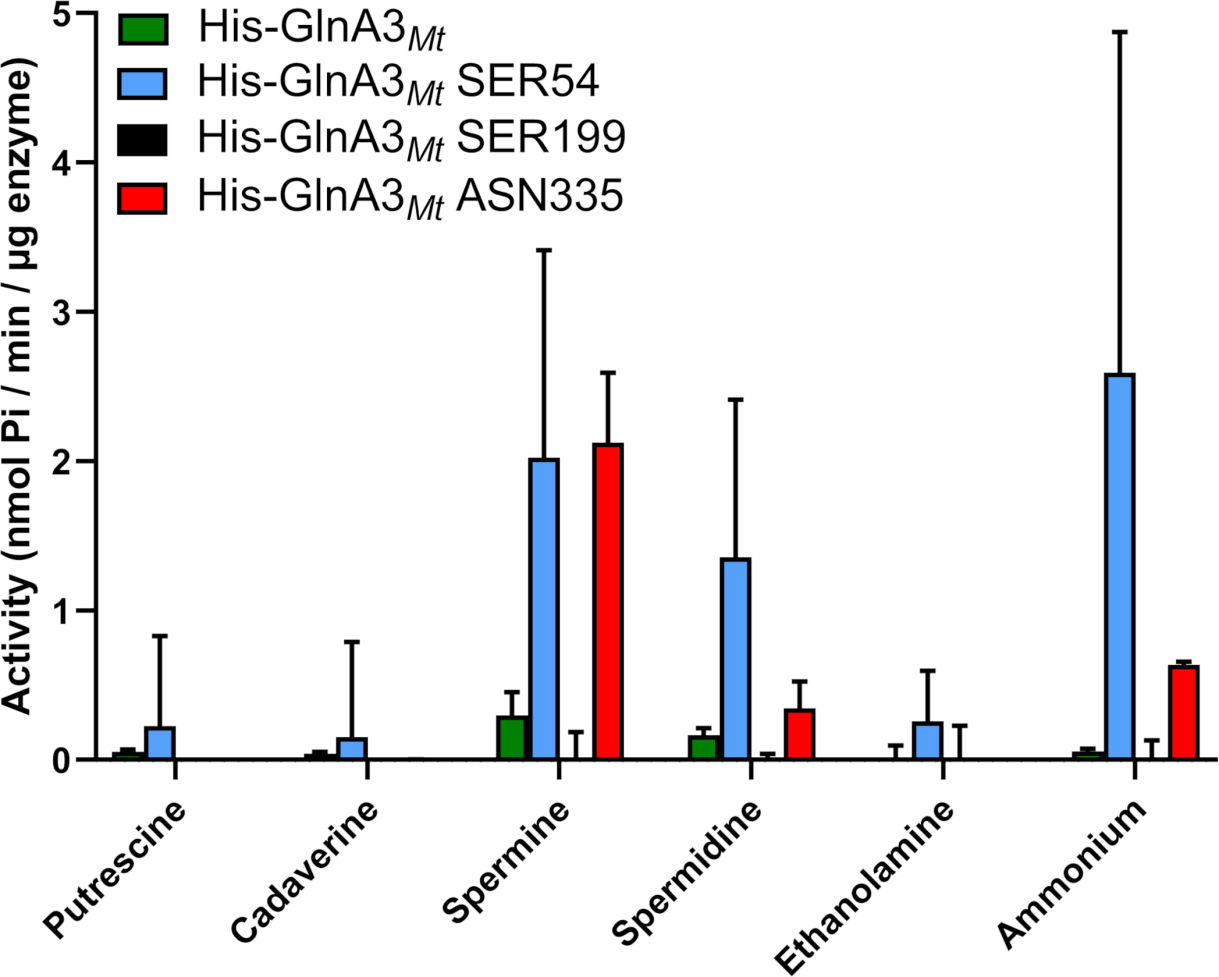
Specific activity of His-Strep-GlnA3*_Mt_* and His-Strep-GlnA3_Mt_* variants with different nitrogen-containing substrates. All substrates were used at a concentration of 50 mM. The mean value of *n* = 3 biological replicates with *n* = 3 technical replicates each with standard error is shown.

Based on these observations, these three amino acids (Ser54, Ser199, Asn335) were mutated via site-directed mutagenesis and the respective recombinant proteins were purified. Conducting activity and substrate specificity testing via the GS-based *in vitro* assay, it was demonstrated that the GlnA3*_Mt_*Ser199 enzyme variant exhibited no activity for any polyamine, in contrast to the WT and GlnA3*_Mt_*Ser54 and GlnA3*_Mt_*Asn335 variants (Fig. 4). These results were further verified by HPLC/MS analysis of reaction products (Fig. S4).

### Spermine is toxic to *M. tuberculosis*

**Fig 5:**
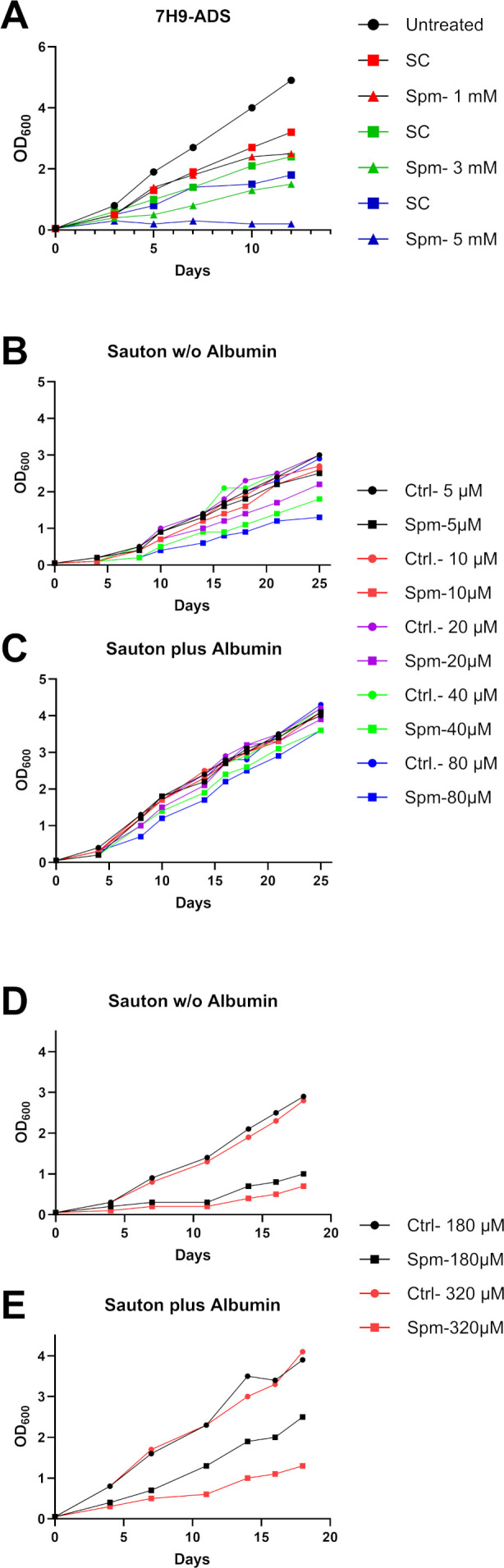
Growth of *M. tuberculosis* in the presence of spermine. Cells were incubated for a period of 14–26 days in (**A**) 7H9 media supplemented with ADS in the presence of 0.5–5 mM spermine (shown in one representative experiment out of four), (**B–E**) Sauton media in the presence of 5–80 µM (**B, C**) or 180–320 µM spermine (**D, E**) either incubated without (w/o) albumin (**B, D**) or with added albumin (**C, E**). SC, solvent control (DMSO); Spm, spermine.

In order to assess the potential toxicity of spermine against *M. tuberculosis*, we first investigated its effect on *M. tuberculosis* in the standard *M. tuberculosis* laboratory culture medium, 7H9, supplemented with albumin, dextrose, and sodium chloride (ADS). Growth curve analyses revealed that 5 mM spermine was required to achieve 90% inhibition (IC_90_) of *M. tuberculosis* growth (Fig. 5A) with 50% inhibition (IC_50_) at 3 mM. Similar values were obtained irrespective of the supplement (ADS or OADC, see Material and Methods) in a fluorescence-based test system employing an *M. tuberculosis* H37Rv strain expressing a red fluorescent protein (Fig. S5A and B).

Since previous studies have shown that spermine conjugates with albumin (39), we investigated the activity of spermine in a minimal medium without supplement (Sauton’s media), and observed that spermine was able to inhibit the growth of *M. tuberculosis* at a substantially lower concentration (IC_90_ of 180–320 µM, IC_50_ of 80 µM) (Fig. 5B) compared to in the albumin-containing 7H9 medium. When albumin was added to the minimal media (3 mg/mL), the growth inhibitory activity of spermine was reduced (Fig. 5C through E). This may likely explain the higher minimum inhibitory concentration (MIC) of spermine in 7H9 standard media containing albumin in the supplement. This also indicates that the presence of serum factors has an impact on the antimicrobial activity of spermine. The latter needs to be considered when evaluating the antimicrobial role of spermine in the infected host.

### The *M. tuberculosis ΔglnA3_Mt_* mutant is not sensitive to spermine

As GlnA3*_Sc_* plays a fundamental role in the survival of *S. coelicolor* at high spermine concentrations (20), we decided to employ a genetic approach to interrogate whether the same is true in *M. tuberculosis*. To this end, we constructed an unmarked, in-frame *glnA3_Mt_*-deletion mutant of *M. tuberculosis*, as described in Materials and Methods (40, 41). The *ΔglnA3_Mt_* (*Δrv1878*) mutant was identified by PCR, and validated by Southern blotting and targeted genome sequencing.

**Fig 6:**
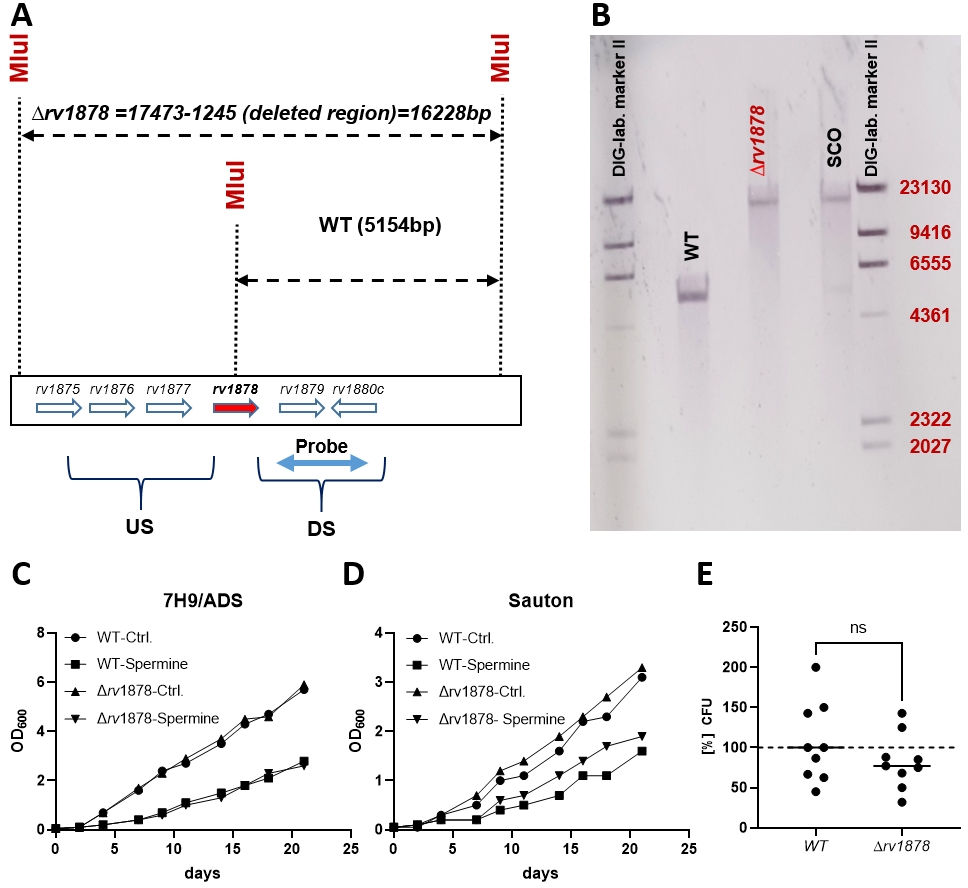
Generation and characterization of the *M. tuberculosis* ∆*glnA3* mutant. (**A and B**) Southern blotting analyses to confirm gene deletion. The protocol was designed such that the wild-type genomic DNA would yield a smaller band (5,154 bp) when digested with the enzyme *Mlu*I and hybridized with the probe flanking the DS (downstream) region, while the mutant DNA would yield a higher molecular weight band (16,228 bp). Genomic DNA from the single cross-over mutant yielded bands of both sizes, as expected. (**C**) Sensitivity of the mutant and wild-type bacteria to 3 mM (1/2 MIC) spermine, as determined by growth curve (OD_600_) analyses in 7H9 media, (**D**) sensitivity of the mutant and wild-type bacteria to 80 µM (1/2 MIC) spermine in Sauton’s media, (**E**) sensitivity of the mutant and wild type to 2 mM spermine, as determined by CFU counts and survival estimation (relative to the untreated control) in Sauton media for 3 h. US, upstream; SCO, single cross over; ns, not significant).

The susceptibility of the mutant to spermine relative to the wild type was first investigated by the broth microdilution method. The MIC was found to be similar to that of the wild type. Next, spermine sensitivity was investigated by monitoring the growth of the strains in the presence of sub-inhibitory concentrations of spermine (half the MIC). Again, no difference in growth rate was observed between the mutant and wild type (Fig. 6C and D). Finally, in order to eliminate the possibility that the difference between the mutant and the wild type was too small to detect with standard growth curve analyses or by MIC determinations, we evaluated the effect of spermine treatment on bacteria by determining colony-forming units (CFU). Three hours of treatment at excess concentrations of spermine (2 mM) in Sauton’s media revealed no difference in CFUs between the tested strains. In summary, all methods revealed that the mutant did not show an elevated sensitivity to spermine under the conditions tested (Fig. 6C through E).

### RNAseq analysis: spermine does not affect *Rv1878* expression*,* but upregulates the efflux pump encoding gene *rv3065*

To investigate if *rv1878 (glnA3_Mt_*) expression is regulated by spermine stress, we initially performed RNAseq analysis of the wild-type bacteria cultured in 7H9 media and treated at half the MIC (3 mM), relative to the untreated control. No significant change in gene expression of *rv1878* was observed (less than twofold difference; Fig. S6A), a result that was validated by reverse-transcriptase quantitative PCR (RT-PCR) data (Fig. S6B). Since albumin could have possibly interfered with the activity of spermine (as discussed above) we repeated the experiment with a medium lacking albumin to apply a more pronounced spermine stress. To this end RNA from bacteria cultured and treated in Sauton’s media was analyzed by RNAseq, however, the regulation of *rv1878* was also not altered by spermine stress in these conditions (Fig. S6C). These data show that GlnA3 mRNA levels in *M. tuberculosis* are minimally affected by spermine stress under the conditions analyzed.

**Fig 7:**
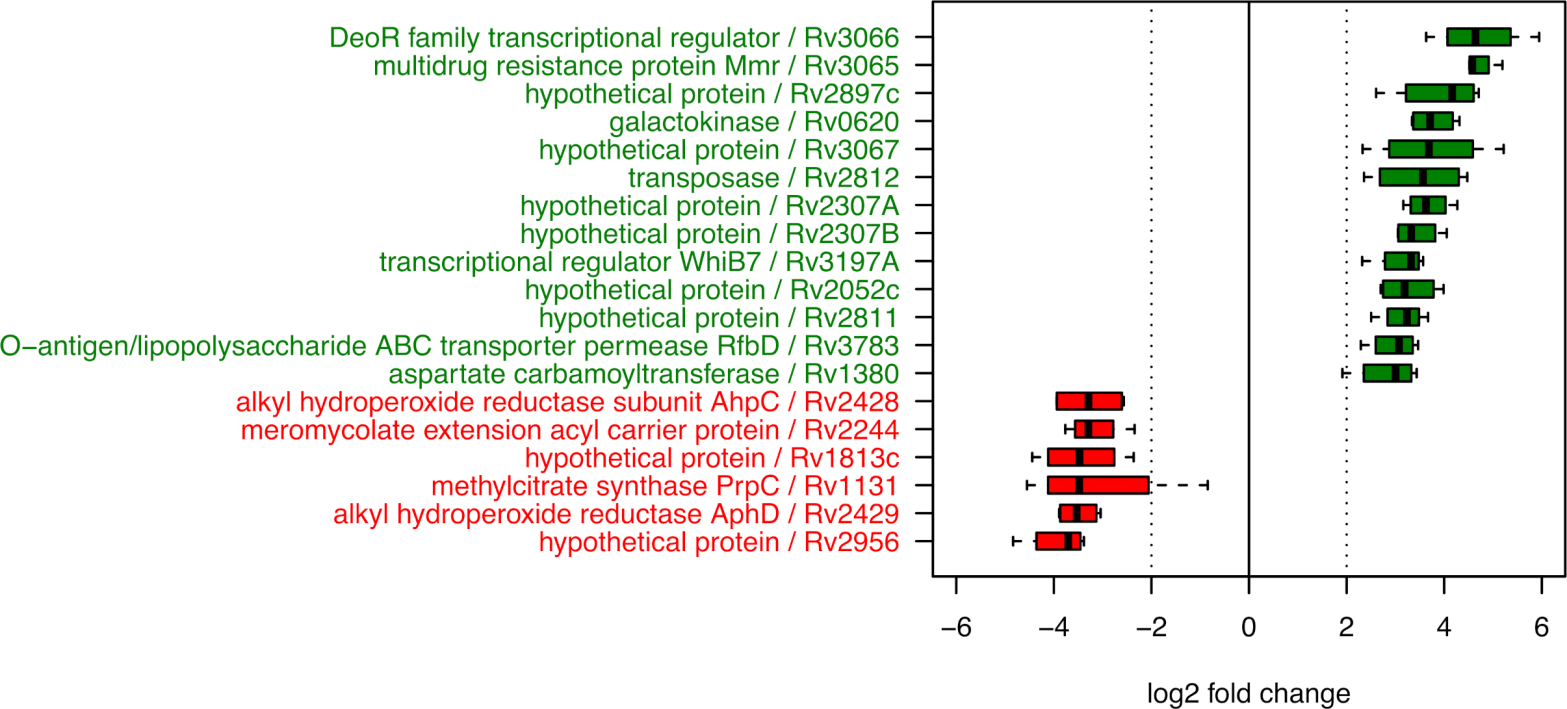
Expression of genes altered upon spermine treatment of *Mycobacterium tuberculosis* wild type in 7H9 media. Transcript levels were determined by RNAseq of bacterial total RNA following treatment. Shown are box plots obtained from four independent experiments.

To investigate which other factors were possibly regulated by spermine stress, we compared the genome-wide differences in gene expression of wild-type *M. tuberculosis* with and without spermine treatment. For six genes a reduced expression level with a log2 median fold change less than −3 was determined. In contrast, for 13 genes with a sufficient number of reads the expression was increased by a log2 median fold change larger than 3 (Fig. 7).

The five genes found to be most prominently upregulated were *rv3066, rv3065, rv2897c, rv0620, and rv3067*. For *rv0620* (log2 median fold change of 3.7; galactokinase), to the best of our knowledge, no specific role in the infection process or polyamine metabolism has been described. *rv2897c* (4.2 log2 fold; hypothetical protein) was previously found upregulated in *M. tuberculosis in vitro* in response to exposure to lung surfactant preparations. Interestingly, the three other highly upregulated genes (*rv3065, rv3066, rv3067*) are all located within the same genetic locus. The gene *rv3065* (log2 median fold change 4.6) codes for Mmr, a multidrug efflux pump that confers resistance to tetraphenylphosphonium (TPP), erythromycin, ethidium bromide, acriflavine, safranin O, pyronin Y, and methyl viologen (42). The gene *rv3066* (log2 median fold change 4.6) codes for a putative transcriptional regulator of the DeoR family, while *rv3067* (log2 median fold change 3.7) encodes an uncharacterized protein, localized in the plasma membrane (https://www.uniprot.org/uniprotkb/I6YB21/entry).

## MATERIALS AND METHODS

### Strains and growth conditions

*M. tuberculosis* (MTB) H37Rv was obtained from ATCC (27294). The auto-fluorescent strain used is an H37Rv derivative transformed with the episomal plasmid pCherry (43, 44). This strain constitutively expresses the fluorescent protein*m*Cherry (a variant of the *Discosoma* sp. red fluorescent protein [*Ds*Red]) (45). Strains were cultured in either Difco Middlebrook 7H9 broth (Becton Dickinson, Heidelberg, Germany) supplemented with either ADS (0.005 g/mL Bovine albumin fraction V, 0.002 g/mL dextrose, 0.85 mg/mL sodium chloride), 0.05% tyloxapol (Sigma Aldrich), and 0.02% (vol/vol) glycerol, or OADC (0.05 mg/mL oleic acid, 0.005 g/mL Bovine albumin fraction V, 0.002 g/mL dextrose, 0.004 mg/mL catalase, 0.85 mg/mL sodium chloride), or cultured in the ready-made Sauton’s media (HIMEDIA) containing glycerol (0.02%) and tyloxapol (0.05%). The solid media used for plating was 7H11 agar-based (Sigma Aldrich) supplemented with either ADS or OADC. Spermine was obtained from Sigma Aldrich, and 100 mM stocks were made in DMSO and stored at −20°C for downstream experiments.

The parental strain *S. coelicolor* M145 as well as all mutants (Table 3) were incubated for 4–5 days at 30°C on the defined Evans-agar base (modified after [46]) supplemented with the following nitrogen sources: 50 mM ammonium chloride, 50 mM L-glutamine, 200 mM putrescine dihydrochloride, 25 mM spermidine trihydrochloride, or 25 mM spermine tetrahydrochloride in appropriate concentrations. Genetic manipulation of *S. coelicolor* M145 was performed as described previously (47, 48).

**TABLE 3:** Strains and plasmids used in this study

Strains	Genotype/phenotype	Reference
*E. coli* NovaBlue	*rec*A1, *end*A1, *gyr*A96, *thi*-1, *hsdR*17 (rK12−, mK12+) *supE*44, *relA*1, *lac* [F', *pro*AB, *lacI*^q^, *lacZ*ΔM15, Tn*10*] (Tet^R^)	Novagen
*E. coli* Novablue pRM4*glnA3*	NovaBlue with the plasmid pRM4*glnA3* (Km^R^)	(49)
*E. coli* XL1-Blue	*rec*A1, *end*A1, *gyr*A96, *thi*-1, *hsd*R17, *sup*E44, *rel*A1, *lac,* [F’, *pro*AB, *lac*I^q^Z, M15Tn*10*, (Tet^R^)]	(50)
*E. coli* XL1-Blue pRM4-*glnA3_Mt_* S54D	XL1-Blue with the plasmid pRM4-*glnA3_Mt_* S54D	This work
*E. coli* XL1-Blue pRM4-*glnA3_Mt_* S199N	XL1-Blue with the plasmid pRM4-*glnA3_Mt_* S199N	This work
*E. coli* XL1-Blue pRM4-*glnA3_Mt_* N335L	XL1-Blue with the plasmid pRM4-*glnA3_Mt_* N335L	This work
*E. coli* S17-1	*recA*, *pro*, *mod*+, *res*−, *tra* genes from plasmid RP4 integrated in the chromosome, donor strain for conjugation	(51)
*E. coli* S17-1 pRM4 *glnA3_Mt_* S54D	S17-1 with the plasmid pRM4-*glnA3_Mt_* S54D, for conjugation with *S. coelicolor* M145	This work
*E. coli* S17-1 pRM4 *glnA3_Mt_* S199N	S17-1 with the plasmid pRM4-*glnA3_Mt_* S199N, for conjugation with *S. coelicolor* M145	This work
*E. coli* S17-1 pRM4 *glnA3_Mt_* N335L	S17-1 with the plasmid pRM4-*glnA3_Mt_* N335L, for conjugation with *S. coelicolor* M145	This work
*E. coli* S17-1 pRM4*glnA3_Mt_*	S17-1 with plasmid pRM4*glnA3_Mt_* for conjugation with *S. coelicolor* M145	This work
*E. coli* BL21 (DE3) pLysS	F−, *ompT*, *hsdFB* (r_B_−m_B_−) *gal dcm* (DE3) pLysS (Cm*^R^*)	(52)
*E. coli* BL21 pET30*glnA3_Mt_*	Bl21 (DE3) pLysS, over-expression of *glnA3_Mt_*	This work
*E. coli* BL21 pET30*glnA3_Mt_*S54D	BL21(DE3) with the plasmid pET30*glnA3_Mt_*S54D	This work
*E. coli* BL21 pET30*glnA3_Mt_*S199N	BL21(DE3) with the plasmid pET30*glnA3_Mt_*S199N	This work
*E. coli* BL21 pET30*glnA3_Mt_*N335L	BL21(DE3) with the plasmid pET30*glnA3_Mt_*N335L	This work
*S. coelicolor* M145	*S. coelicolor A3(2*) without native plasmids: *spc1^−^* and *spc2*^−^	(48)
*S. coelicolor* M145 *∆glnA3*	*glnA3* mutant strain of *S. coelicolor* M145; insertional inactivation of *glnA3* by an *aac*(3)IV cassette, (Apr^R^)	(25)
*S. coelicolor* M145 ∆*glnA3* pRM4*glnA3_Mt_*	*glnA3* mutant strain of *S. coelicolor* M145 with pRM4*glnA3_Mt_*; (Apr^R^ and Km^R^)	This work
*S. coelicolor* M145 ∆*glnA3* pRM4*glnA3_Mt_*S54D	*S. coelicolor ∆glnA3* with the plasmid pRM4*glnA3_Mt_*S54D	This work
*S. coelicolor* M145 ∆*glnA3* pRM4*glnA3_Mt_*S199N	*S. coelicolor ∆glnA3* with the plasmid pRM4*glnA3_Mt_*S199N	This work
*S. coelicolor* M145 ∆*glnA3* pRM4*glnA3_Mt_*N335L	*S. coelicolor ∆glnA3* with the plasmid pRM4*glnA3_Mt_*N335L	This work
*E. coli* BL21 pYT/his-*glnA3*	pYT9 derivative for overexpression of His-*glnA3_Sc_*	(20)
*M. tuberculosis* H37Rv	Wild type	(53)
*M. tuberculosis* H37Rv *∆glnA3*	*M. tuberculosis* derivative with deleted *glnA3* gene	This work
**Plasmids**		
pRM4	pSET152*ermE*p* with artificial RBS	(54)
pRM4*glnA3*	pRM4-derivative with *glnA3_Sc_* (*S. coelicolor*)	(55)
pRM4*glnA3_Mt_*	pRM4-derivative with *glnA3_Mt_* (*M. tuberculosis*)	This work
pET30 Ek/LIC	Ligation independent cloning (LIC), T7 Promoter, T7 transcription start, phage f1 origin of replication, detachable N-terminal His-tag and S-tag, C-terminal His-tag, T7 terminator, *lacI* coding sequence, pBR322 *ori*	Novagen
pET30*glnA3_Mt_*	pET30-derivative with *glnA3_Mt_* (*M. tuberculosis*)	This work
pK18	pUC-derivative, *lacZ’α-* complementation system, Km^R^	(52)

### Growth curve and susceptibility testing of *M. tuberculosis*

Frozen stocks were used to inoculate starter cultures and after 7–10 days of incubation, these were used to sub-culture into the relevant test growth conditions. For each tested concentration of spermine, a corresponding, untreated, DMSO control was included. The OD_600_ was measured and recorded every second day. To independently validate the OD_600_ data, *M. tuberculosis* growth was monitored by fluorescence measurements using a mCherry expressing *M. tuberculosis* H37Rv strain which was analyzed at various time points, in a 96-well dark plate, using a multimode plate reader (Synergy 2, Agilent) (56). The MIC of test compounds was determined with the broth-microdilution method, using resazurin to enable colorimetric detection of growth as previously described (57, 58). For CFU enumeration, logarithmic phase cultures were adjusted to an OD_600_ of 0.002 in fresh culture medium, (approximately 10^4^ CFU/mL), and exposed to either 2 mM spermine or an equivalent volume of DMSO for 3 h in a 96-well plate, at 37°C. Culture aliquots were then serially diluted and plated on solid media, followed by CFU counting after 3 weeks of incubation.

### Generation and complementation of the *M. tuberculosis* ∆*glnA3* mutant

Gene knockout of *glnA3* in *M. tuberculosis* was achieved as previously described (40, 41). Briefly, approximately 2,500 bp of genomic DNA directly upstream (US) and downstream (DS) of *rv1878* (*glnA3*) were amplified using pfu high fidelity GC-rich target polymerase (Agilent). Primers are listed in Table 4. PCR products were subsequently cloned into the pJET sub-cloning vector (CloneJET PCR Cloning Kit, Thermofisher). The US and DS fragments were then transferred from the pJET constructs to the p2NIL suicide plasmid (40), using restriction enzyme-ligation methodology (using enzymes *Bsr*GI, S*pe*I, and *Hin*dIII). Then the *Pac*I restriction fragment containing, *lacZ*, and *sacB* genes was restricted from pGOAL17 (40) and cloned into the *Pac*I site of p2NIL. The phage-resistant and endonuclease I (*endA1*) deficient *E. coli* strain D10-beta (New England Biolabs Inc.) was used in the entire cloning process. The integrity of the inserts was verified by Sanger sequencing at the end of each cloning step throughout the cloning process (sequencing primers found in Table 4). The resulting construct p2NIL-A3-US/DS-G17, was used to transform *M. tuberculosis*, and the mutant was generated through two subsequent homologous recombination events as previously described (40). The mutant was screened by PCR (primers in Table 4), confirmed by Southern blotting as previously described (38), and by targeted genome sequencing (59). The mutant was complemented as previously described (41), using the pMVhsp60 integrative vector (60), where *glnA3* was cloned downstream of the heat shock promoter (P_hsp60_), to maintain constitutive expression at 37°C .

**TABLE 4:** Primers used in the study

Primer	Sequence	RS	Purpose	Product/size
A3-USF	GCTGTACATCTCAAGCCAGGAATCGTCAT	BsrgI	In-frame deletion of 1,245 bp of the 1,353 bp of *rv1878*	US = 2,402 bp
A3-USR	GCACTAGTCAAGCGGTGTGGCTGTCAT	SpeI		
A3-DSF	GCACTAGTTCGCGGTACGCCAGTTAGA	SpeI		DS = 2,404 bp
A3-DSR	GCAAGCTTAACCGCGCCAACTACCTGAC	HindIII		
A3-SF	GGTCGACTCCCTCGCAGTTT	NA	Screening of deletion mutants	
A3-SR1	CGATCCGGCTCAGTGTTCC	NA		Set 1: WT = 614 bp ∆ = 0 bp Set 2: WT = 2,251 bp ∆ =2,251 – 1,245 = 1,006 bp
A3-SR2	GCAGCAGAACCCGATCCTGT	NA		
A3-F	AAGCTTAGAAGGAGAAGTACCGATGACAGCCACACCGCTTG	HindIII	Complementation of ∆*rv*1878	1383 + 16 + 6 + 8 = 1,413
A3-R	GCGTTAACTTACACACTCCAAGCCATCCGG	HpaI		
G17-NIL-DS-R	GTGCCTGACTGCGTTAGCAA	NA	Sequencing the final construct	5,000–6,000 bp Flanking outside inserted US and DS
NIL-LACZ-US-F1	GCACCGCCGAAACCCTTAT	NA	Sequencing the final construct	
NIL-SACB-US-F2	GGCTGCAGGAATTCGATATCA	NA	Sequencing the final construct	
A3-Seq-R	AACGATGGTTTGGCCGGT		Sequencing inserts of the *rv1878* deletion construct The homologous recombination template	
A3-Seq-F1	GCCAGGAATCGTCATGTGC			
A3-Seq-F2	CAGCACCGTTGACCAGTCGT			
A3-Seq-F3	CAGTGGCGCTGGGTTTCTT			
A3-Seq-F4	AGTGCCATCTCCGGCTGTCT			
A3-Seq-F5	TTGAAGCGACAGCCAGACC			
A3-Seq-F6	TTGATCCGCAGACTTATTGGG			
A3-Seq-F7	AATCGTGTTGCTGCACTGCT			
A3-Seq-F8	CGTTCGATGTCAAAGCGGT			
A3-Seq-F9	CCTACGACGTCCACAATCCC			
78-seq-3	GCATCGCTATCGAGCAGTT		Sequencing of insert in *rv1878* complementation construct (primers MV-F and SR-1 were used as well)	
78-seq-4	GGAATCTATGCATGCTGGG			

Reverse transcriptase PCR primers and probes				
Primer	Sequence	Probe	Sequence (5′-Fam/3’-quencher)	Purpose
16S-RT-F	GACCACGGGATGCATGTC	16S-probe	CACCCCACCAACAAGCTGATAGGC	Housekeeping gene to normalize RT-PCR data of each gene
16S-RT-R	CCGTCGTCGCCTTGGTAG			
76-RT-F	GCTATCAACCAATACTTTCTGCACT	76-probe	TCGACGAAATGCGGCACG	Quantification of the expression level of *Rv1876*
76-RT-R	ATCCAGCAACAAGATGCGA			
77-RT-F	GCTGTACCTGGTCGTCCT	77-probe	TGTTCTGCACGATGAGAACGAGCCAC	Quantification of the expression level of *Rv1877*
77-RT-R	AAGGTCACACCCGATGTTG			
78-RT-F	GATACGCCGGACCAACACA	78-probe	ATCCTGGCCTCGGCGCCA	Quantification of the expression level of *Rv1878*
78-RT-R	ACAGAAGGTATGCCACACCG			
79-RT-F	CGACTGGCTGGTGGAG	79-probe	TCGTGAGCGTGGCTGCCCG	Quantification of the expression level of *Rv1879*
79-RT-R	GGCGCAGTATCTCGTTG			
ovglnA3mtF	GACGACGACAAGATGACAGCCACACCGCTTGC			Amplification of the gene *glnA3_Mt_* of *M. tuberculosis* H37Rv for pET30 vector
ovglnA3mtR	GAGGAGAAGCCCGGTTACACACTCCAA GCCAT			Amplification of the gene *glnA3_Mt_* of *M. tuberculosis* H37Rv for pET30 vector
Km1	GACAGGATGAGGATCGTTTC			
Km2	AATCTCGTGATGGCAGGTTG			
T7	TAATACGACTCACTATAGGG		Eurofins Scientific SE	Sequencing of insert in pET30*glnA3_Mt_*
T7 term	CTAGTTATTGCTCAGCGGT		Eurofins Scientific SE	Sequencing of insert in pET30*glnA3_Mt_*
CglnA3mt-F	CGCCCATATGACAGCCACACCGC			Amplification of the gene *glnA3_Mt_* of *M. tuberculosis* H37Rv for pRM4 vector
CglnA3mt-R	TCCAAGCTTTTACACACTCCAAGCCA			Amplification of the gene *glnA3_Mt_* of *M. tuberculosis* H37Rv for pRM4 vector
glnA3mtmut4F	GGCGCCAACCCGGTGTGGCATACCTTC TGTATCGAC			
glnA3mtmut4R	CACCGGGTTGGCGCCGAGGCCAGGATTGGCGAATGT			
glnA3mtmut5F	GAGATCAACTTAGCGCCGCAGCCGCCGGTCGCGGCC			
glnA3mtmut5R	CGCTAAGTTGATCTCGAATTGGTTGGCACCGTATTC			
glnA3mtmut6F	GGCGGGCTCGTGGAGGTGAAGGTCGTCGACCCGTCG			
glnA3mtmut6R	CTCCATGAGCCCGCCGTACGCGCTGCCAGCCCCGCC			
hrdB-qrt1	TGACCAGATTCCGGCCACTC			PCR-primer for housekeeping gene *hrdB* (control)
hrdB-qrt2	CTTCGCTGCGACGCTCTTTC			PCR-primer for housekeeping gene *hrdB* (control)
M13 uni (−21)	TGTAAAACGACGGCCAGT			Sequencing of insert in pRM4*glnA3_Mt_*
M13 rev (−49)	GAGCGGATAACAATTTCACACAGG			Sequencing of insert in pRM4*glnA3_Mt_*
pRM_GenF	CTGCAAGGCGATTAAGTTGG			Sequencing of insert in pRM4*glnA3_Mt_*
pRM_GenR	TTATGCTTCCGGCTCGTATG			Sequencing of insert in pRM4*glnA3_Mt_*

### Complementation of the *S. coelicolor ∆glnA3* mutant

For complementation of the *S. coelicolor glnA3_Sc_* mutant, the *glnA3_Mt_* gene from *M. tuberculosis* was amplified by PCR using *CglnA3mtF* and *CglnA3mtR* primers and cloned into the multiple cloning site of pRM4 plasmid between the *Nde*I and *Hin*dIII restriction sites downstream of the constitutive erythromycin promoter *ermE*p*. The kanamycin resistance cassette was amplified by PCR using the pK18 plasmid as a template and primers *aphIIupperEcoR*I and *aphIIlowerHind*III, and introduced into the multiple cloning site of the recombinant plasmid pRM4-*glnA3MtC* between the *Eco*RI and *Hin*dIII restriction sites. The construct was confirmed by colony PCR as well as by sequencing and introduced into the *S. coelicolor glnA3_Sc_* mutant by biparental conjugation using *E. coli* S-17. Clones were selected based on their kanamycin and apramycin resistance phenotypes. The correct integration of the pRM4-*glnA3Mt* was confirmed by PCR and sequencing.

### Cloning, expression, and purification of His-Strep-GlnA3*_Mt_*

The GlnA3*_Mt_* encoding gene *rv1878* was amplified using the genomic DNA of *M. tuberculosis* as a template. It was inserted into the expression vector pET-30 Ek/LIC (Novagen) under the control of the IPTG inducible T7 promoter using the pET-30 Ek/LIC Cloning Kit (Novagen). Recombinant His/Strep-GlnA3_Mt_ was produced in *E. coli* BL21 (DE3). Initially, cells were incubated overnight at 37°C in LB medium, then transferred to fresh LB medium and incubated at 25°C until the culture reached an optical density of 0.5 at 600 nm. Subsequently, the 1 L culture was induced with 1 mM IPTG and incubated at 25°C overnight. His/Strep-GlnA3_Mt_ was purified by nickel ion affinity chromatography essentially as recommended by the resin manufacturer (GE-Healthcare) as described in reference (20). Purified His/Strep-GlnA3_Mt_ was dialyzed against 20 mM Tris, and 100 mM NaCl (pH 8.0), or used for further purification steps. His/Strep-Tag was cleaved using enterokinase according to the protocol of the manufacturer (NEB) and GlnA3*_Mt_* was immediately purified from the digestion mix by size-exclusion chromatography as directed by the resin manufacturer (GE-Healthcare).

### HPLC/ESI-MS detection of a glutamylated product

For the detection of the glutamylated product of the GlnA3*_Mt_* catalyzed reaction, an HPLC/ESI-MS procedure was applied. Standard reactions contained 20 mM HEPES (pH 7.2), 10 mM ATP, 150 mM glutamate sodium monohydrate, 150 mM putrescine dihydrochloride, cadaverine dihydrochloride, spermidine trihydrochloride, or spermine tetrahydrochloride, and 20 mM MgCl_2_. Reactions were initiated by the addition of 10 µg of purified His/Strep-GlnA3*_Mt_* or GlnA3*_Mt_* (or no enzyme as a control) and incubated at 30°C for 5 min. The reaction mixture was then incubated at 100°C for 5 min in order to stop the reaction.

HPLC/ESI-MS analysis was performed on an Agilent 1200 HPLC series using a Reprosil 120 C_18_ AQ column, 5 µm, 200 mm by 2 mm fitted with a pre-column 10 mm by 2 mm (Dr. Maisch GmbH, Ammerbuch, Germany) coupled to an Agilent LC/MSD Ultra Trap System XCT 6330 (Agilent, Waldbronn, Germany). For analysis the following conditions were used: 0.1% formic acid as solvent A and acetonitrile with 0.06% formic acid as solvent B at a flow rate of 0.4 mL min^−1^. The gradient was as follows: t_0_ = t_5_ = 0% B, t_20_ = 40% B (time in minutes). The injection volume was 2.5 µL, and the column temperature was 40°C. ESI ionization was done in negative mode with a capillary voltage of 3.5 kV and a drying gas temperature of 350°C. Standard solutions of polyamines were prepared with distilled water. HPLC detection limit for different polyamines was 0.1 mM.

### Modified GS activity assay

The enzymatic activity of GlnA3_Mt_ variants was tested in a modified GS activity assay as described in reference (28). The absorbance was measured at 655 nm using a Microplate reader. Raw absorbance readings were put into Excel (Microsoft).

### *In silico* protein modeling and docking studies

To build the *in silico* models for the different GlnA-like enzymes an existing template from the Protein Data Bank (PDB) was selected. The initial template search was done using the template search function of SWISS-MODEL (30) by inputting of the amino acid sequence in FASTA format, plain text, or UniProtKB accession code. SWISS-MODEL was then performing a search for evolutionary-related protein structures against the SWISS-MODEL template library SMTL (61) and using database search methods BLAST, and HHblits (62, 63). The resulting template structures were ranked and further evaluated by SWISS-MODEL using the estimated Global Model Quality Estimate (GMQE) (61) and the Quaternary Structure Quality Estimate (QSQE) (64). Top-ranked templates and alignments were compared to verify whether they represent alternative conformational states or cover different regions of the target protein. In such cases, multiple templates were selected automatically and different models were built accordingly (30). Out of the resulting list of possible templates different templates were chosen based on the GMQE, QSQE, identity, and oligomeric state. Also, value was given to select templates out of different bacterial phyla to get quality control with diversity. Using the selected templates as a base SWISS-MODEL built a 3D protein model estimating the real 3D structure of the protein. Therefore, SWISS-MODEL started with the conserved atom coordinates defined by the target-template alignment and then coordinated residues corresponding to insertions/deletions in the alignment that were generated by loop modeling, and a full-atom protein model was obtained by constructing the non-conserved amino acid side chains (61). SWISS-MODEL used the ProMod3 modeling engine and the OpenStructure computational structural biology framework (65). The evaluation of the build 3D protein models was done using the QMEAN scoring function, the MolProbity score, and a Ramachandran plot of the model. The QMEAN score provided an estimate of the “degree of nativeness” of the structural features observed in a model and described the likelihood that a given model was of comparable quality compared to experimental structures (35). The MolProbility score relied heavily on the power and sensitivity provided by optimized hydrogen placement and all-atom contact analysis, complemented by updated versions of covalent geometry and torsion-angle criteria (37). The Ramachandran plot plotted the torsion angles of the different amino acids against each other to verify the correct folding of the *in silico* model (36). Based on the highest QMEAN score and the Ramachandran plot the best *in silico* 3D protein models were chosen. Molecule structures were obtained from the PubChem database. Molecular docking was performed using UCSF Chimera software.

### Site-directed mutagenesis

To select candidate amino acids for mutagenesis the *in silico* 3D structures of GlnA3*_Mt_* were compared with the template structures they originated from. The principal 3D model of the GlnA3*_Mt_* enzyme was based on the structure of GlnA1 (PDB code 1HTO) of *M. tuberculosis* (31). Therefore, the 3D structural model of GlnA3*_Mt_* and the template structure were opened with SWISS-PDB viewer (66), PyMol (PyMOL, The PyMOL Molecular Graphics System, Version 2.0 Schrödinger, LLC), or UCSF Chimera. Through literature research, key amino acids of the template could be identified and then marked in the 3D template structure. The amino acids that overlap directly with the key amino acids of the template were marked. The site-directed mutagenesis approach used was based on the protocol described by Zheng et al. (67). It employed a PCR approach with specifically designed primers to introduce the required base-pair changes into the glnA3 coding sequence. The GlnA3_Mt_* mutated variants were generated using the pET-30 Ek/LIC plasmid with the cloned *glnA3* gene as a template and were expressed in *E. coli* strain BL21 (DE3).

### RNA sample preparation

A 10 mL volume of early to mid-logarithmic phase cultures was treated with either spermine or DMSO alone and harvested 3 h later using the RNA Pro Blue kit (MP Bio). The resuspended cells were homogenized using the Fast Prep Homogenizer (30 s, 6 m/s, four times, 5 min intermittent). The cell lysate was filtered twice using PTFE syringe filters (13 mm diameter, 0.2 µM pores size) and taken out of the BSL3 laboratory for further purification. The first purification was performed using the Direct-zol RNA Miniprep Plus (R2070, 100 µg binding capacity) including an in-column DNA digestion step, according to the manufacturer’s instructions. The purified samples were quantified using a spectrophotometer and diluted if the concentration exceeded 200 ng/µL. Then they were further digested using the Turbo DNA-free kit (Thermo Fisher) according to the manufacturer’s instructions. Each sample was treated with Turbo DNAse in two consecutive rounds, to ensure complete DNA digestion. The digested samples were further purified and concentrated using the RNA clean and concentrator kit-25 (R1017, 50 µg binding capacity; Zymo Research), including another in-column DNA digestion step, according to the manufacturer’s instructions. The resulting samples were used for Next Generation RNA sequencing performed by Eurofins Genomics GmbH.

### RNA sequencing

Bulk RNA sequencing of the resulting samples as well as expression quantification was performed by Eurofins Genomics GmbH. RNA quality was measured using the Agilent 2100 Bioanalyzer and all but one sample had RIN values > 8. Sequencing was performed on the Illumina Novaseq 6000 using the NEBNext(R) Ultra II Directional RNA Library Prep Kit, generating between 13.5 and 17.3 million 150 bp paired-end read pairs. Reads were mapped to the genome sequence of *M. tuberculosis* H37Rv (NC_000962.3; obtained from https://www.ncbi.nlm.nih.gov/nuccore/NC_000962.3?report = fasta) using bwa-mem version 0.7.12-r1039 (68). With the exception of one sample with 89% reads mapping to H37Rv, all samples had a mapping rate larger than 97%. Reads were subsequently counted for 3,906 *Mycobacterium tuberculosis* H37Rv genes according to RefSeq annotation (obtained via https://www.ncbi.nlm.nih.gov/Taxonomy/Browser/wwwtax.cgi?id = 83332) using tool featureCounts (69). Raw overall read counts per sample are between 4.4 and 8.8 million (counting each read pair once and omitting multi-mapping and low-quality reads). Trimmed mean of M-values normalization (70) of raw counts was performed using the edgeR package version 3.16.5 (71). In a multidimensional scaling plot, the first dimension separated spermine-treated from untreated samples. Normalized counts were used for the computation of fold changes and the computation of median fold changes, which were log2 transformed whenever specifically noted.

#### RT-PCR

In order to perform the reverse-transcriptase quantitative PCR (RT-PCR), RNA samples were converted to cDNA using the Maxima First Strand cDNA kit (Thermo Fisher). The reverse transcribed samples were run in 10 µL reactions on a LightCycler 480, using the LightCycler 480 master mix. Quantification was carried out by the integrated software of the LightCycler, according to a probe-based assay (labeled at the 5’-end with FAM and at the 3’-end with a quencher), using *M. tuberculosis* genomic DNA to generate a standard curve (10–1,000 pg/µL). The probe and primers were designed by TIB MOLBIOL Syntheselabor GmbH. Various sets of primers were designed and tested in order to obtain the final optimized set that was used for quantification (Table 3).

## DISCUSSION

During evolution, many intracellular pathogens (including protozoan parasites) have developed strategies to access selected nutrients from the host for their growth and survival. *M. tuberculosis* is an example of an intracellular pathogen, which is very well adapted to survive within a hostile macrophage environment. The long-term persistence of the pathogen within the host is in part a consequence of a subtle equilibrium between the nutritive needs of host and pathogens. On one hand, the presence of high intracellular polyamine levels in macrophages (72) may lead to growth inhibition or cell death of *M. tuberculosis* (73). On the other hand, polyamines may provide a nutrient source that can be exploited by *M. tuberculosis*.

Since free spermine is toxic to cells, it has to be immediately excreted or metabolically detoxified by modifications such as acetylation (74) or gamma glutamylation (20, 21). Spermine modified in such a manner shows reduced toxicity toward bacteria due to loss of the net positive charge (75). Glutamylated spermine may be subsequently utilized as an N/C source or excreted from cells (4).

In order to investigate how *M. tuberculosis* can survive in high polyamine concentrations, we took advantage of the knowledge on polyamine metabolism in *S. coelicolor,* a model actinobacterium that shares essential features in nitrogen metabolism with *M. tuberculosis*. The gamma-glutamylpolyamine synthetase GlnA3*_Sc_* is the primary enzyme of the polyamine utilization pathway in *S. coelicolor* (20) catalyzing the first step of polyamine utilization, the detoxification of polyamine by glutamylation. Thus, we hypothesized that a similar polyamine utilization pathway might enable pathogenic actinobacteria, such as *M. tuberculosis*, to colonize host cells and evade the host’s immune response by conferring resistance against toxic polyamines and ensuring putative polyamine utilization necessary for long-term persistence. Proof of such a pathway in *M. tuberculosis* could also provide a novel target for the development of antitubercular drugs. Transcription of *glnA3_Mt_*, the mycobacterial orthologue of *glnA3_Sc_*, has previously been reported in broth culture (76) and the respective protein has been detected in a guinea pig model of tuberculosis *in vivo*, during the chronic stage of this disease (77, 78). However, both transposon site hybridization and CRISPRi-based transcriptional repression approaches have shown that *glnA3*/*rv1878* is not an essential or vulnerable gene in *M. tuberculosis* for *in vitro* growth in 7H9 broth culture (79, 80). However, a given essentiality or vulnerability always depends on the conditions or the microenvironment the bacteria faces. Although its presence was considered to be not essential for bacterial homeostasis in 7H9 broth cultures, its role was not previously evaluated in the presence of high polyamine concentrations reflecting disease-relevant conditions.

In the current study, we observed that *M. tuberculosis* growth can be inhibited by spermine. Using a GS-based *in vitro* enzymatic activity assay we showed that GlnA3*_Mt_* (Rv1878) acts as a gamma-glutamylspermine synthetase by generating glutamylated spermine from spermine, glutamate, and ATP. In an *in vitro* phosphate release assay we demonstrated that purified recombinant GlnA3*_Mt_* prefers spermine as a substrate, over putrescine, cadaverine, spermidine, or other monoamines and amino acids. Interestingly, genetic complementation with GlnA3*_Mt_* rescued the growth defect of an *S. coelicolor* Δ*glnA3_Sc_* mutant grown on putrescine, although this enzyme has low substrate specificity toward short-chain polyamines (see Fig. 2). These results led to the conclusion that GlnA3*_Mt_* plays a specific role in the detoxification of the polyamine spermine, similar to the function of its homolog GlnA3*_Sc_* in *S. coelicolor*. We therefore hypothesized that GlnA3*_Mt_* may be essential for the survival of *M. tuberculosis* during spermine stress, which would make this enzyme a potential target for antitubercular chemotherapeutic development.

Interestingly, the deletion of the *glnA3* gene in *M. tuberculosis* did not result in immediate cell death or a reduced growth rate of the strain in the presence of high spermine concentrations. Thus, additional factors seem to be involved in polyamine detoxification in *M. tuberculosis*. Our RNAseq analysis of polyamine-treated *M. tuberculosis* revealed several genes to be significantly upregulated in response to spermine, with one gene locus being of particular interest: the *rv3065-3067* operon. *rv3065* encodes an efflux pump suggesting that active excretion of polyamines is the bacterium’s primary defense against this class of molecules. Of note, bioinformatic analyses and previous studies (42) revealed that these proteins are not found in either *Mycobacterium smegmatis* or in *S. coelicolor*. Rv3065 was the first mycobacterial protein identified and described in the small membrane protein family in *M. tuberculosis* (81). This represents a group of efflux pumps that contain four transmembrane domains and confer resistance to aromatic dyes, derivatives of TPP, and quaternary amines (82). Rv3065 has independently been shown to mediate the efflux of different chemical compounds and antibiotics belonging to the pyrrole and pyrazolone chemical classes, which represent nitrogen-containing heterocyclic, aromatic compounds (83). Joint transcription of *rv3065-rv3067* has previously been observed in *M. tuberculosis* treated with thioridazine (84). Also, over-expression of *rv3065* in *M. tuberculosis* (85) and *M. smegmatis* (81) increased resistance to cationic TPP, erythromycin, ethidium bromide, acriflavine, safranin O, and pyronin Y. Thus it is not unlikely that polycationic molecules such as spermine may also be transported by Rv3065.

Such a resistance mechanism may render GlnA3_Mt_-based detoxification of spermine less important and could explain the survival of the *glnA3_Mt_* mutant in the presence of spermine. These findings suggest that besides GlnA3_Mt_ other factors need to be considered if antitubercular compounds are to be designed and developed to target polyamine/spermine detoxification in *M. tuberculosis*.

### Conclusion

In this study, the enzymatic function of GlnA3*_Mt_* and the anti-mycobacterial activity of spermine were investigated. The spermine detoxification mechanisms in *M. tuberculosis,* as well as an interplay of GlnA3 and potential polyamine transporters like Rv3065, seem to constitute a complex system that enables the bacterium to avoid spermine-induced toxicity. Further studies are needed to investigate the function of other genes involved in polyamine metabolism in *M. tuberculosis*.

## SUPPLEMENTAL MATERIAL

Supplemental tables and figures jb.00439-24-s0001.docx Tables S1 to S4; Fig. S1 to S7.

ASM does not own the copyrights to Supplemental Material that may be linked to, or accessed through, an article. The authors have granted ASM a non-exclusive, world-wide license to publish the Supplemental Material files. Please contact the corresponding author directly for reuse.
