# Supplementary material for: GlnA3Mt is able to glutamylate spermine but it is not essential for the detoxification of spermine in Mycobacterium tuberculosis
Source: J Bacteriol. 2025 Jan 30;207(2):e00439-24. doi: 10.1128/jb.00439-24 (PMC11841054; doi:10.1128/jb.00439-24)
Supplement: Supplemental tables and figures — Tables S1 to S4; Fig. S1 to S7. [file jb.00439-24-s0001.docx]

| **Tab. 1. Physiological role of the *glnA3_Mt_* gene product in the *S. coelicolor* *glnA3* mutant grown in the presence of polyamines and other nitrogen sources.** |
| --- |
| \| **Medium** \| **M145  (WT)** \| ***∆glnA3_Sc_*** \| ***∆glnA3_Sc_***  ***+glnA3_Mt_*** \| ***∆glnA3_Sc_***  ***+glnA3_Sc_*** \| \| --- \| --- \| --- \| --- \| --- \| \| **LB-Agar** \| +++ \| +++ \| +++ \| +++ \| \| **R5-Agar** \| +++ \| +++ \| +++ \| +++ \| \| **Evans-Agar  + NH_4_Cl (50 mM)** \| +++ \| +++ \| +++ \| +++ \| \| **Evans-Medium**  **+ Glutamine (50 mM)** \| +++ \| +++ \| +++ \| +++ \| \| **Evans-Medium**  **+ Putrescine (200 mM)** \| +++ \| - \| + \| ++ \| \| **Evans-Medium**  **+ Spermidine (25 mM)** \| +++ \| - \| + \| ++ \| \| **Evans-Medium**  **+ Spermine (25 mM)** \| +++ \| - \| + \| ++ \| |
| Note: +: poor growth, ++: moderate growth, +++: strong growth; -: no growth. |

| **Tab. 2. Superposition of residues in GlnA3*_Sc_* and GlnA3*_Mt_***   \| GlnA3*_Sc_* \| E151 \| E153 \| E207 \| E214 \| H263 \| N260 \| G261 \| R316 \| H265 \| R339 \| W327 \| A169 \| \| --- \| --- \| --- \| --- \| --- \| --- \| --- \| --- \| --- \| --- \| --- \| --- \| --- \| \| GlnA3*_Mt_* \| E137 \| E139 \| E190 \| E197 \| H246 \| S243 \| G244 \| R299 \| H248 \| R322 \| A310 \| none \| |
| --- | --- | --- | --- | --- | --- | --- | --- | --- | --- | --- | --- | --- | --- | --- | --- | --- | --- | --- | --- | --- | --- | --- | --- | --- | --- | --- |

**Tab. 3. Strains and plasmids used in this study.**

| **Strains** | Genotype/Phenotype | Reference |
| --- | --- | --- |
| *E. coli* NovaBlue | *rec*A1, *end*A1, *gyr*A96, *thi*-1, *hsdR*17 (rK12-, mK12+) *supE*44, *relA*1, *lac* [F', *pro*AB, *lacI*^q^, *lacZ*ΔM15, Tn*10*] (Tet^R^) | Novagen |
| *E. coli* Novablue pRM4*glnA3* | NovaBlue with the plasmid pRM4*glnA3* (Km^R^) | (79) |
| *E. coli* XL1-Blue | *rec*A1, *end*A1, *gyr*A96, *thi*-1, *hsd*R17, *sup*E44, *rel*A1, *lac,* [F’, *pro*AB, *lac*I^q^Z, M15Tn*10*, (Tet^R^)] | (80) |
| *E. coli* XL1-Blue pRM4-*glnA3_Mt_* S54D | XL1-Blue with the plasmid  pRM4-*glnA3_Mt_* S54D | This work |
| *E. coli* XL1-Blue pRM4-*glnA3_Mt_* S199N | XL1-Blue with the plasmid  pRM4-*glnA3_Mt_* S199N | This work |
| *E. coli* XL1-Blue pRM4-*glnA3_Mt_* N335L | XL1-Blue with the plasmid  pRM4-*glnA3_Mt_* N335L | This work |
| *E. coli* S17-1 | *recA*, *pro*, *mod*+, *res*−, *tra* genes from plasmid RP4 integrated in the chromosome, donor strain for conjugation | (81) |
| *E. coli* S17-1 pRM4 *glnA3_Mt_* S54D | S17-1 with the plasmid  pRM4-*glnA3_Mt_* S54D, for conjugation with *S. coelicolor* M145 | This work |
| *E. coli* S17-1 pRM4 *glnA3_Mt_* S199N | S17-1 with the plasmid  pRM4-*glnA3_Mt_* S199N, for conjugation with *S. coelicolor* M145 | This work |
| *E. coli* S17-1 pRM4 *glnA3_Mt_* N335L | S17-1 with the plasmid  pRM4-*glnA3_Mt_* N335L, for conjugation with *S. coelicolor* M145 | This work |
| *E. coli* S17-1 pRM4*glnA3_Mt_* | S17-1, with plasmid pRM4*glnA3_Mt_* for conjugation with *S. coelicolor* M145 | This work |
| *E. coli* BL21 (DE3) pLysS | F−, *ompT*, *hsdFB* (r_B_−m_B_−) *gal dcm* (DE3) pLysS (Cm*^R^*) | (82) |
| *E. coli* BL21 pET30*glnA3_Mt_* | Bl21 (DE3) pLysS, over-expression of *glnA3_Mt_* | This work |
| *E. coli* BL21 pET30*glnA3_Mt_*S54D | BL21(DE3) with the plasmid pET30*glnA3_Mt_*S54D | This work |
| *E. coli* BL21 pET30*glnA3_Mt_*S199N | BL21(DE3) with the plasmid pET30*glnA3_Mt_*S199N | This work |
| *E. coli* BL21 pET30*glnA3_Mt_*N335L | BL21(DE3) with the plasmid pET30*glnA3_Mt_*N335L | This work |
| *S. coelicolor* M145 | *S. coelicolor A3(2)* without native plasmids: *spc1^-^* and *spc2*^-^ | (48) |
| *S. coelicolor* M145 *∆glnA3* | *glnA3* mutant strain of *S. coelicolor* M145; insertional inactivation of *glnA3* by an *aac*(3)IV cassette, (Apr^R^) | (25) |
| *S. coelicolor* M145 ∆*glnA3* pRM4*glnA3_Mt_* | *glnA3* mutant strain of *S. coelicolor* M145 with pRM4*glnA3_Mt_*; (Apr^R^ and Km^R^) | This work |
| *S. coelicolor* M145 ∆*glnA3* pRM4*glnA3_Mt_*S54D | *S. coelicolor* *∆glnA3* with the plasmid pRM4*glnA3_Mt_*S54D | This work |
| *S. coelicolor* M145 ∆*glnA3* pRM4*glnA3_Mt_*S199N | *S. coelicolor* *∆glnA3* with the plasmid pRM4*glnA3_Mt_*S199N | This work |
| *S. coelicolor* M145 ∆*glnA3* pRM4*glnA3_Mt_*N335L | *S. coelicolor* *∆glnA3* with the plasmid pRM4*glnA3_Mt_*N335L | This work |
| *E. coli* BL21 pYT/his-*glnA3* | pYT9 derivative for overexpression of His-*glnA3_Sc_* | (20) |
| *M. tuberculosis* H37Rv | wild type | (83) |
| *M. tuberculosis* H37Rv *∆glnA3* | *M. tuberculosis* derivative with deleted *glnA3* gene | This work |
| **Plasmids** |  |  |
| pRM4 | pSET152*ermE*p* with artificial RBS | (84) |
| pRM4*glnA3* | pRM4-derivative with *glnA3_Sc_* (*S. coelicolor*) | (85) |
| pRM4*glnA3_Mt_* | pRM4-derivative with *glnA3_Mt_* (*M. tuberculosis*) | This work |
| pET30 Ek/LIC | ligation independent cloning (LIC), T7 Promoter, T7 transcription start, phage f1 origin of replication, detachable N-terminal His-tag and S-tag, C-terminal His-tag, T7 terminator, *lacI* coding sequence, pBR322 *ori* | Novagen |
| pET30*glnA3_Mt_* | pET30-derivative with *glnA3_Mt_* (*M. tuberculosis*) | This work |
| pK18 | pUC-derivative, *lacZ’α-*  complementation system, Km^R^ | (82) |

**Table 4. Primers used in the study**

| **Primer** | **Sequence** | **RS*** | **Purpose** | **Product/ size** |
| --- | --- | --- | --- | --- |
| **A3-USF** | \|  \| \| --- \|   GCTGTACATCTCAAGCCAGGAATCGTCAT | BsrgI | In frame deletion of 1245 bp of the 1353 bp of *rv1878* | US= 2402 bp |
| **A3-USR** | GCACTAGTCAAGCGGTGTGGCTGTCAT | SpeI |  |  |
| **A3-DSF** | GCACTAGTTCGCGGTACGCCAGTTAGA | SpeI |  | DS= 2404 bp |
| **A3-DSR** | GCAAGCTTAACCGCGCCAACTACCTGAC | HindIII |  |  |
| **A3-SF** | GGTCGACTCCCTCGCAGTTT | NA | Screening of deletion mutants |  |
| **A3-SR1** | CGATCCGGCTCAGTGTTCC | NA |  | **Set 1:** WT= **614bp**  **∆ = 0 bp**  **Set 2:** WT=**2251bp**  ∆ = 2251-1245= **1006bp** |
| **A3-SR2** | GCAGCAGAACCCGATCCTGT | NA |  |  |
| **A3-F** | AAGCTTAGAAGGAGAAGTACCGATGACAGCCACACCGCTTG | HindIII | Complementation of ∆*rv*1878 | 1383+16+6+8=1413 |
| **A3-R** | GCGTTAACTTACACACTCCAAGCCATCCGG | HpaI |  |  |
| **G17-NIL-DS-R** | GTGCCTGACTGCGTTAGCAA | NA | Sequencing the final construct | 5000-6000bp  Flanking outside inserted US and DS |
| **NIL-LACZ-US-F1** | GCACCGCCGAAACCCTTAT | NA | Sequencing the final construct |  |
| **NIL-SACB-US-F2** | GGCTGCAGGAATTCGATATCA | NA | Sequencing the final construct |  |
|  |  |  |  |  |
| **A3-Seq-R** | AACGATGGTTTGGCCGGT |  | Sequencing inserts of the *rv1878* deletion construct  The homologous recombination template |  |
| **A3-Seq-F1** | GCCAGGAATCGTCATGTGC |  |  |  |
| **A3-Seq-F2** | CAGCACCGTTGACCAGTCGT |  |  |  |
| **A3-Seq-F3** | CAGTGGCGCTGGGTTTCTT |  |  |  |
| **A3-Seq-F4** | AGTGCCATCTCCGGCTGTCT |  |  |  |
| **A3-Seq-F5** | TTGAAGCGACAGCCAGACC |  |  |  |
| **A3-Seq-F6** | TTGATCCGCAGACTTATTGGG |  |  |  |
| **A3-Seq-F7** | AATCGTGTTGCTGCACTGCT |  |  |  |
| **A3-Seq-F8** | CGTTCGATGTCAAAGCGGT |  |  |  |
| **A3-Seq-F9** | CCTACGACGTCCACAATCCC |  |  |  |
| **78-seq-3** | GCATCGCTATCGAGCAGTT |  | Sequencing of insert in *rv1878* complementation construct (primers MV-F and SR-1 were used as well) |  |
| **78-seq-4** | GGAATCTATGCATGCTGGG |  |  |  |
| **Reverse transcriptase PCR primers and probes** | | | | |
| **Primer** | **Sequence** | **Probe** | **Sequence (5’-Fam/ 3’-quencher)** | **Purpose** |
| 16S-RT-F | GACCACGGGATGCATGTC | 16S-probe | CACCCCACCAACAAGCTGATAGGC | Housekeeping gene to normalise RT-PCR data of each gene |
| 16S-RT-R | CCGTCGTCGCCTTGGTAG |  |  |  |
| 76-RT-F | GCTATCAACCAATACTTTCTGCACT | 76-probe | TCGACGAAATGCGGCACG | Quantification of the expression level of *Rv1876* |
| 76-RT-R | ATCCAGCAACAAGATGCGA |  |  |  |
| 77-RT-F | GCTGTACCTGGTCGTCCT | 77-probe | TGTTCTGCACGATGAGAACGAGCCAC | Quantification of the expression level of *Rv1877* |
| 77-RT-R | AAGGTCACACCCGATGTTG |  |  |  |
| 78-RT-F | GATACGCCGGACCAACACA | 78-probe | ATCCTGGCCTCGGCGCCA | Quantification of the expression level of *Rv1878* |
| 78-RT-R | ACAGAAGGTATGCCACACCG |  |  |  |
| 79-RT-F | CGACTGGCTGGTGGAG | 79-probe | TCGTGAGCGTGGCTGCCCG | Quantification of the expression level of *Rv1879* |
| 79-RT-R | GGCGCAGTATCTCGTTG |  |  |  |
| ovglnA3mtF | GACGACGACAAGATGACAGCCACACCGCTTGC |  |  | Amplification of the gene *glnA3_Mt_* of *M. tuberculosis* H37Rv for pET30 vector |
| ovglnA3mtR | GAGGAGAAGCCCGGTTACACACTCCAA GCCAT |  |  | Amplification of the gene *glnA3_Mt_* of *M. tuberculosis* H37Rv for pET30 vector |
| Km1 | GACAGGATGAGGATCGTTTC |  |  |  |
| Km2 | AATCTCGTGATGGCAGGTTG |  |  |  |
| T7 | TAATACGACTCACTATAGGG |  | Eurofins Scientific SE | Sequencing of insert in pET30*glnA3_Mt_* |
| T7 term | CTAGTTATTGCTCAGCGGT |  | Eurofins Scientific SE | Sequencing of insert in pET30*glnA3_Mt_* |
| CglnA3mt-F | CGCCCATATGACAGCCACACCGC |  |  | Amplification of the gene *glnA3_Mt_* of *M. tuberculosis* H37Rv for pRM4 vector |
| CglnA3mt-R | TCCAAGCTTTTACACACTCCAAGCCA |  |  | Amplification of the gene *glnA3_Mt_* of *M. tuberculosis* H37Rv for pRM4 vector |
| glnA3mtmut4F | GGCGCCAACCCGGTGTGGCATACCTTC TGTATCGAC |  |  |  |
| glnA3mtmut4R | CACCGGGTTGGCGCCGAGGCCAGGATTGGCGAATGT |  |  |  |
| glnA3mtmut5F | GAGATCAACTTAGCGCCGCAGCCGCCGGTCGCGGCC |  |  |  |
| glnA3mtmut5R | CGCTAAGTTGATCTCGAATTGGTTGGCACCGTATTC |  |  |  |
| glnA3mtmut6F | GGCGGGCTCGTGGAGGTGAAGGTCGTCGACCCGTCG |  |  |  |
| glnA3mtmut6R | CTCCATGAGCCCGCCGTACGCGCTGCCAGCCCCGCC |  |  |  |
| hrdB-qrt1 | TGACCAGATTCCGGCCACTC |  |  | PCR-primer for housekeeping gene *hrdB* (control) |
| hrdB-qrt2 | CTTCGCTGCGACGCTCTTTC |  |  | PCR-primer for housekeeping gene *hrdB* (control) |
| M13 uni (-21) | TGTAAAACGACGGCCAGT |  |  | Sequencing of insert in pRM4*glnA3_Mt_* |
| M13 rev (-49) | GAGCGGATAACAATTTCACACAGG |  |  | Sequencing of insert in pRM4*glnA3_Mt_* |
| pRM_GenF | CTGCAAGGCGATTAAGTTGG |  |  | Sequencing of insert in pRM4*glnA3_Mt_* |
| pRM_GenR | TTATGCTTCCGGCTCGTATG |  |  | Sequencing of insert in pRM4*glnA3_Mt_* |

**Supplementary figures**

|  |
| --- |
| Suppl. Fig. 1. Reaction catalyzed by gamma-glutamylpolyamine synthetase with indicated substrates, products and molecular weight of each molecule. |

| 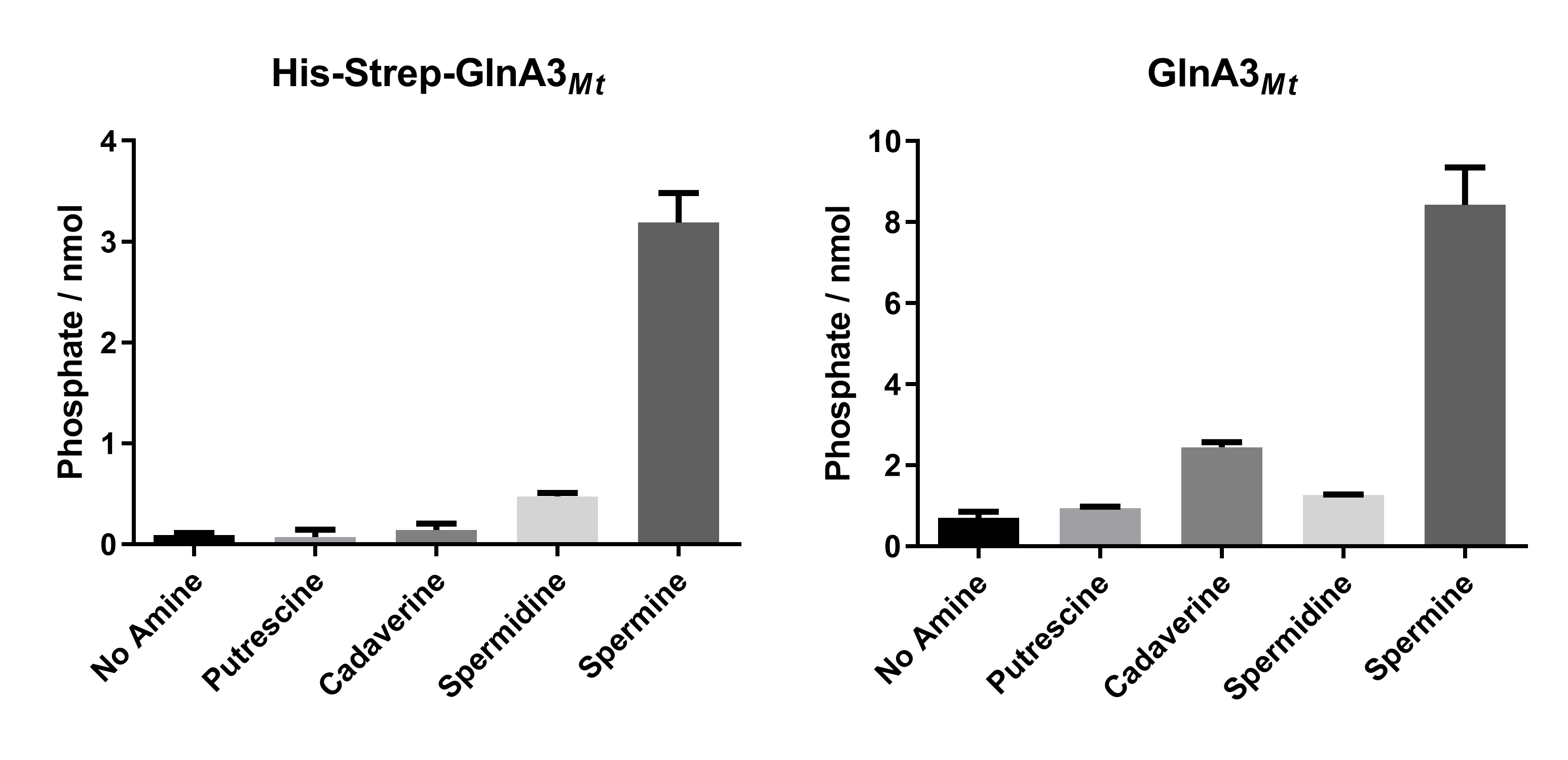 |
| --- |
| Suppl. Fig. 2. Specific activity of the native GlnA3*_Mt_* with different polyamines. All substrates were tested at a concentration of 50 mM. The mean value of minimum n=3 biological replicates with n=3 technical replicates each with standard error is shown. |

| 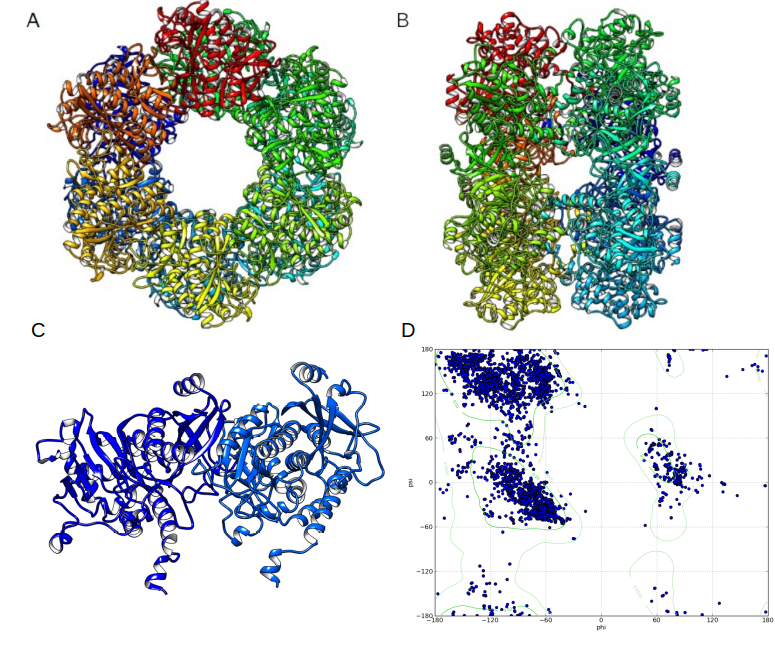 |
| --- |
| Suppl. Fig. 3. Structural alignment of the 3D model structure of GlnA3*_Mt_*, based on and superposed with the GlnA*_Mt_* template (PDB code 1BVC (29)). A) each of the twelve subunits is marked in a different color (color scheme “chain”), B) side-view of two 6-unit containing rings, C) a representation of the active site and substrate binding pocket, D) Ramachandran plot demonstrating the quality of the generated GlnA3*_Mt_* model, based on visualization of energetically allowed regions for backbone dihedral angles ψ against φ of amino acid residues in the structure.  C |

| 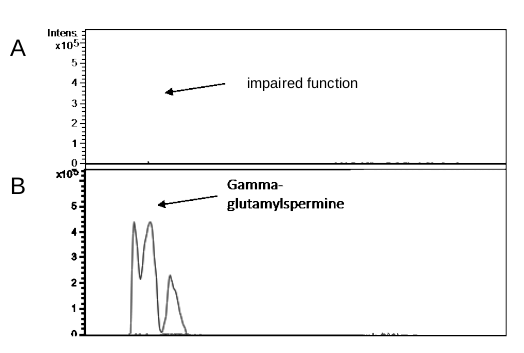 |
| --- |
| Suppl. Fig. 4. HPLC/ESI-MS analysis of His-Strep-GlnA3*_Mt_*SER199 and His-Strep-GlnA3*_Mt_*. Two samples were analyzed in MS negative mode: reaction mixtures with addition of His-Strep-GlnA3*_Mt_*SER199 (A) and with addition of His-Strep-GlnA3*_Mt_* (B). Extracted ion chromatograms for the His-Strep-GlnA3*_Mt_* reaction product corresponding to gamma-glutamylspermine with charge to mass ratio of *m/z* 331 was shown (B), and no product in the sample with GlnA3*_Mt_*SER199 was detected (A). |

| 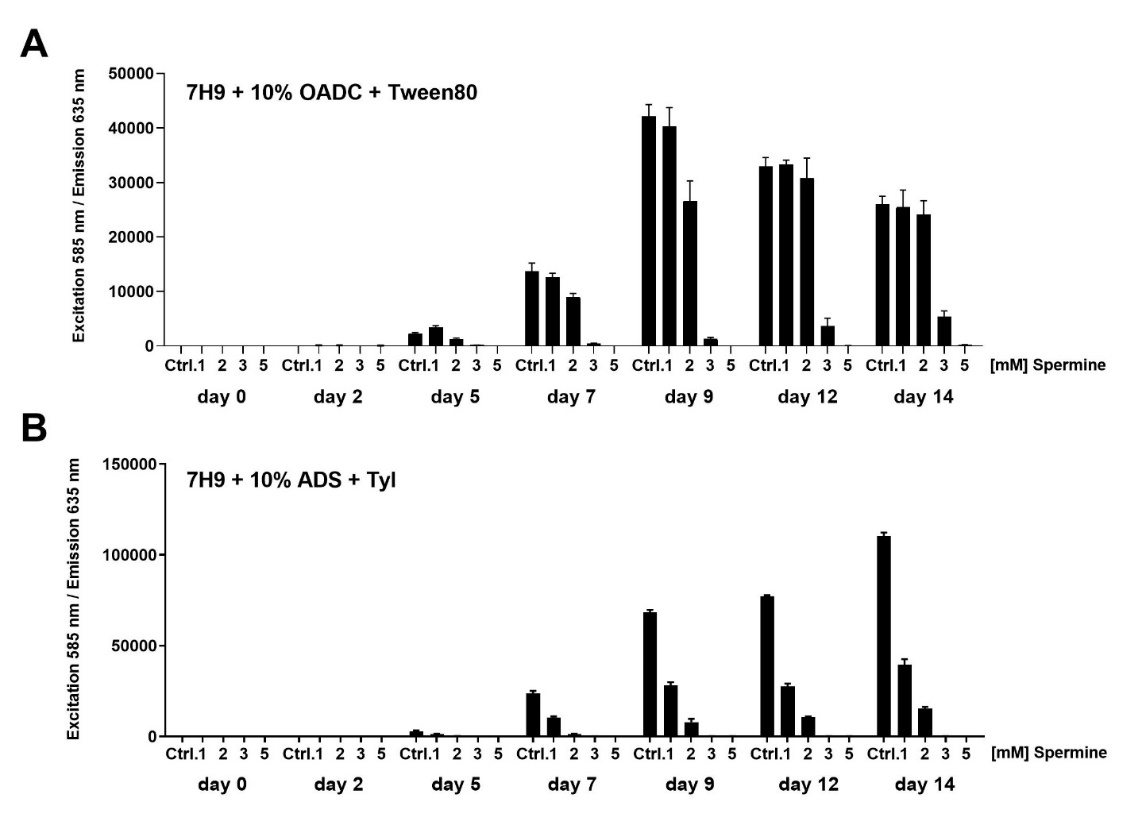 |
| --- |
| Suppl. Fig. 5. Growth of *M. tuberculosis* in the presence of spermine. *m*Cherry- expressing Mtb H37Rv bacteria (55) were incubated for a period of 14 days in the absence or presence of spermine in (A) 7H9 medium + 10% Oleic acid-Albumin-Dextrose-Catalase (OADC) + Tween80 (0,05%) or (B) in 7H9 medium + Albumin-Dextrose-Sodium chloride (ADS) + Tyloxapol (Tyl) and analyzed as described (55); Ctrl: DMSO; Spm: spermine. Concentrations tested were from 1 mM to 5 mM. |

| 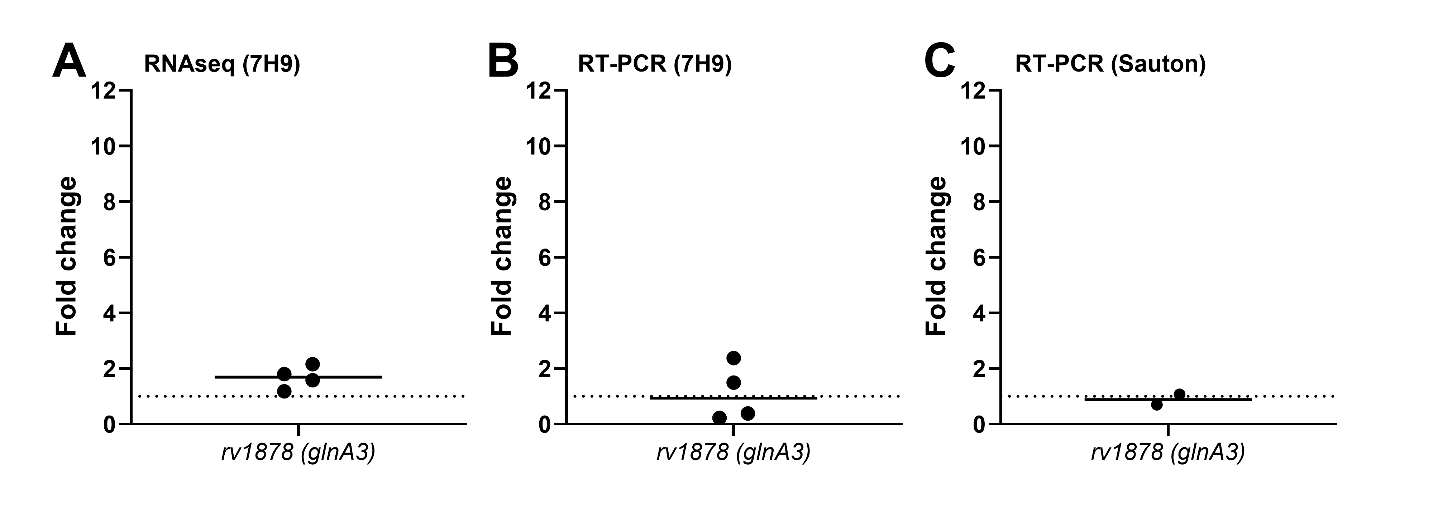 |
| --- |
| Suppl. Fig. 6. Expression level of *rv1878/ GlnA3* in *M. tuberculosis* WT treated with Spermine at half the MIC, relative to the untreated control. The expression was determined by RNAseq (A) and RT-PCR analysis (B, C) in 7H9 (A, B) and Sauton´s (C) medium. |

**Figure legends**

Fig. 1. Purification of GlnA3*_Mt_* and generation of glutamylated spermine and spermidine by GlnA3*_Mt_* in an *in vitro* assay. A) purification of GlnA3*_Mt_* by size-exclusion chromatography (SEC). SEC supported the putative dodecameric quaternary structure of the enzyme. A commercial apoferritin standard was used as a control for molecular mass estimation (elution profile shown in part). B) HPLC/ESI-MS chromatograms demonstrating the presence or absence of glutamylated putrescine, spermidine and spermine in a sample without (1) and with His-Strep-tagged GlnA3*_Mt_* enzyme (2-4). Gamma-glutamylputrescine (m/z 217) (2), gamma-glutamylspermidine (m/z 274) (3), gamma-glutamylspermine (m/z 331) (4). Reaction containing recombinant GlnA3*_Mt_* following removal of the Strep-His affinity tag, gamma-glutamylspermine (m/z 331) (5).

Fig. 2. Specific activity of His-GlnA3*_Mt_* and His-GlnA3*_Sc_* with different nitrogen containing substrates. All substrates were used at 50 mM. The mean value of n=3 biological replicates with n=3 technical replicates each with standard error is shown.

Fig. 3 A) Structural alignment of the 3D model structure of the GlnA3*_Mt_* enzyme (sub-units A and B, the active site in-between). Based on and superposed with the GlnA*_Mt_* template (PDB code 1BVC (27). Superposition of the GlnA3*_Sc_* (violet) and GlnA3*_Mt_* (blue) models with depicted key amino acids identified in GlnA3*_Sc_* (green, from (16) and GlnA3*_Mt_* (red) (see Tab. 2). B) Constellation of amino acid residues required for polyamine substrate binding in GlnA3*_Mt_* (red)*.* C) The residues selected for site-directed mutagenesis i.e. Ser54, Ser199 and Asn335 (cyan) are depicted in the active site of GlnA3*_Mt_* among key residues in the binding pocket. D) structural alignment of structure models of two sub-units of GlnA3*_Mt_* (red and orange) with the docked molecule of spermine (black) in the best scored dock position overlaid with the crystal structure of PauA7*_Pa_* (violet).

Fig. 4. Specific activity of His-Strep-GlnA3*_Mt_* and His-Strep-GlnA3*_Mt_** variants with different nitrogen-containing substrates. All substrates were used at a concentration of 50 mM. The mean value of n=3 biological replicates with n=3 technical replicates each with standard error is shown.

Fig. 5. Growth of *M. tuberculosis* in the presence of spermine. Cells were incubated for a period of 14-26 days in **(A)** 7H9 media supplemented with ADS in the presence of 0.5-5 mM spermine (shown is one representative experiment out of four), **(B-E)** Sauton media in the presence of 5-80 µM (B, C) or 180-320 µM spermine (D, E) either incubated without (w/o) albumin (B, D) or with added albumin (C, E). SC: solvent control (DMSO) ; Spm: spermine

Fig. 6. Generation and characterization of the *M. tuberculosis* ∆*glnA3* mutant. A and B) Southern blotting analyses to confirm gene deletion. The protocol was designed such that the wild type genomic DNA would yield a smaller band (5,154 bp) when digested with the enzyme *Mlu*I and hybridized with the probe flanking the DS (downstream) region, while the mutant DNA would yield a higher molecular weight band (16,228 bp). Genomic DNA from the single cross-over mutant yielded bands of both sizes, as expected. C) Sensitivity of the mutant and wild type bacteria to 3 mM (1/2 MIC) spermine, as determined by growth curve (OD_600_) analyses in 7H9 media, D) Sensitivity of the mutant and wild-type bacteria to 80 µM (1/2 MIC) spermine in Sauton’s media, E) Sensitivity of the mutant and wild type to 2 mM spermine, as determined by CFU counts and survival estimation (relative to the untreated control) in Sauton media for 3 hours. US, upstream; SCO Single cross over; ns, not significant)

Fig. 7. Expression of genes altered upon spermine treatment of *Mycobacterium tuberculosis* wild type in 7H9 media. Transcript levels were determined by RNAseq of bacterial total RNA following treatment. Shown are box plots obtained from 4 independent experiments.
